# Endoplasmic Reticulum Stress and Its Role in Metabolic Reprogramming of Cancer

**DOI:** 10.3390/metabo15040221

**Published:** 2025-03-24

**Authors:** Salvatore Zarrella, Maria Rosaria Miranda, Verdiana Covelli, Ignazio Restivo, Sara Novi, Giacomo Pepe, Luisa Tesoriere, Manuela Rodriquez, Alessia Bertamino, Pietro Campiglia, Mario Felice Tecce, Vincenzo Vestuto

**Affiliations:** 1 Department of Pharmacy, University of Salerno, Via G. Paolo II, 84084 Fisciano, Italy; s.zarrella3@studenti.unisa.it (S.Z.); mmiranda@unisa.it (M.R.M.); snovi@unisa.it (S.N.); gipepe@unisa.it (G.P.); abertamino@unisa.it (A.B.); pcampiglia@unisa.it (P.C.); tecce@unisa.it (M.F.T.); 2 NBFC, National Biodiversity Future Center, 90133 Palermo, Italy; 3 Department of Pharmacy, University of Naples Federico II, Via Domenico Montesano, 49, 80131 Napoli, Italy; verdiana.covelli@unina.it (V.C.); manuela.rodriquez@unina.it (M.R.); 4 Department of Biological, Chemical and Pharmaceutical Sciences and Technologies, University of Palermo, Via Archirafi 28, 90123 Palermo, Italy; ignazio.restivo@unipa.it (I.R.); luisa.tesoriere@unipa.it (L.T.)

**Keywords:** metabolomics, endoplasmic reticulum stress, unfolded protein response, biochemical pathways, cancer, tumor microenvironment, drug discovery

## Abstract

**Background/Objectives:** Endoplasmic reticulum (ER) stress occurs when ER homeostasis is disrupted, leading to the accumulation of misfolded or unfolded proteins. This condition activates the unfolded protein response (UPR), which aims to restore balance or trigger cell death if homeostasis cannot be achieved. In cancer, ER stress plays a key role due to the heightened metabolic demands of tumor cells. This review explores how metabolomics can provide insights into ER stress-related metabolic alterations and their implications for cancer therapy. **Methods:** A comprehensive literature review was conducted to analyze recent findings on ER stress, metabolomics, and cancer metabolism. Studies examining metabolic profiling of cancer cells under ER stress conditions were selected, with a focus on identifying potential biomarkers and therapeutic targets. **Results:** Metabolomic studies highlight significant shifts in lipid metabolism, protein synthesis, and oxidative stress management in response to ER stress. These metabolic alterations are crucial for tumor adaptation and survival. Additionally, targeting ER stress-related metabolic pathways has shown potential in preclinical models, suggesting new therapeutic strategies. **Conclusions:** Understanding the metabolic impact of ER stress in cancer provides valuable opportunities for drug development. Metabolomics-based approaches may help identify novel biomarkers and therapeutic targets, enhancing the effectiveness of antitumor therapies.

## 1. Introduction

The National Cancer Institute defines cancer as “a disease in which some of the body’s cells grow uncontrollably and spread to other parts of the body”. This condition can manifest in almost any tissue of the body, and depending on the specific type and stage, cancer cells can invade surrounding tissues and metastasize to distant organs, further complicating treatment.

Cancer is a complex and heterogeneous group of diseases that constitutes one of the most significant public health challenges, globally affecting millions of individuals each year. Rather than being a singular condition, cancer encompasses over 100 distinct types, including solid tumors, such as breast, lung, and colorectal carcinomas, and hematological malignancies like leukemia and lymphoma. Each subtype is characterized by unique genetic mutations, epigenetic alterations, and differential cellular behaviors, which complicate diagnosis, treatment, and prognosis [1].

Tumorigenesis is generally understood as a multi-step process, beginning with the occurrence of an oncogenic mutation in a single somatic cell. Mutations that enhance growth competitiveness and drive cancer evolution are referred to as cancer driver mutations. Identifying these driver mutations and understanding their roles in tumors are crucial areas of interest in cancer genome research. Recent advancements in sampling and sequencing technologies have improved the detection of somatic mutations and clonal expansion in normal tissues. However, it is now recognized that mutations alone are insufficient for tumor development, and additional molecular events must also occur [2,3,4,5,6].

Various environmental and systemic factors have been epidemiologically confirmed as cancer risk factors, including chemical exposures, unhealthy metabolic behaviors, infections from specific pathogens, and aging. These factors lead to diverse changes at both global and local levels, inducing genetic and epigenetic alterations in transformed cells and significantly affecting the microenvironmental components that predispose individuals to tumor initiation [7]. Among these influences, metabolic regulation emerges as a crucial aspect, acting at the intersection of genetic and environmental modifications.

In particular, cellular metabolism is regulated by both the intrinsic metabolic properties of the cell and the uptake of external nutrients. Tumors alter their metabolic patterns to evade nutrient restrictions and meet their high demands for growth and aberrant proliferation. Additionally, tumors produce oncogenic metabolites that regulate gene and protein expression to promote tumor progression. This metabolic reprogramming is one of the central features of cancer development and progression and it begins early, even in the pre-cancerous phase. Indeed, biochemical alterations are often viewed primarily as adaptive responses to cellular transformation, linked to cancerous conditions. Throughout the processes of cancer development and progression, cancer cells must continuously adjust their metabolic pathways in response to changes in the tumor microenvironment (TME). This includes adaptations to factors such as restricted vascularization, limited availability of nutrients, increased extracellular matrix (ECM) deposition, and the expansion of immune infiltrates and cancer-associated fibroblasts (CAFs). These metabolic adaptations are essential for the growth, proliferation, and metastatic potential of the tumor [8].

Consequently, numerous molecular mechanisms, both intrinsic and extrinsic, converge to modify fundamental cellular metabolism, responding to the three primary requirements of proliferating cells: rapid ATP production to sustain energy levels, increased biosynthesis of macromolecules, and tight regulation of the cellular redox state. To fulfill these requirements, cancer cells undergo metabolic adaptations affecting all four major classes of macromolecules: carbohydrates, proteins, lipids, and nucleic acids [9].

During metabolic reprogramming of malignant tumors, wherein cancer cells alter their metabolic pathways to meet the demands of rapid growth and proliferation, one of the most prominent is a shift from oxidative phosphorylation to aerobic glycolysis, known as the Warburg effect. This shift enables elevated levels of ATP production and the generation of vital metabolic intermediates essential for biosynthetic processes [10].

This metabolic shift is not merely a byproduct of tumor growth but appears to be an adaptive response to spatial and physiological constraints encountered during early tumor development. Oxygen levels drop significantly in these regions, while glucose remains relatively available due to its smaller molecular size and higher diffusion coefficient [11]. Smallbone et al. hypothesized that this diffusion-limited microenvironment imposes strong selective pressures that drive the emergence of the glycolytic phenotype. Using a hybrid cellular automaton model that integrates spatial geometry, cellular metabolism, and environmental gradients, they simulated the evolution of premalignant cells through three key adaptive phases: adaptation to hypoxia via the constitutive upregulation of glycolysis, acidification of the local environment due to lactate accumulation, and the selection of cells resistant to acid-induced toxicity. Their model demonstrates that this sequence of adaptations promotes invasiveness, as cells capable of proliferating in an acidic microenvironment gain a competitive advantage over their less-adapted neighbors [12].

Cancer metabolism has traditionally been described by the Warburg effect, wherein cancer cells predominantly rely on aerobic glycolysis for energy production, even in the presence of sufficient oxygen. However, emerging evidence supports an alternative metabolic model, termed the “reverse Warburg effect”, which underscores the metabolic interplay between CAFs and cancer cells. In this model, CAFs undergo aerobic glycolysis, producing high levels of lactate and pyruvate. These metabolites are then taken up by cancer cells and utilized in oxidative phosphorylation to support tumor growth and survival. This metabolic coupling is facilitated by reactive oxygen species (ROS) generated by cancer cells, which induce metabolic reprogramming in CAFs [10]. This interplay highlights the adaptive plasticity of tumor microenvironments and their ability to exploit stromal cells to meet energetic and biosynthetic demands. Key studies have demonstrated that the reverse Warburg effect is associated with increased stromal expression of glycolytic enzymes, such as lactate dehydrogenase (LDH) and monocarboxylate transporters (MCTs), which mediate lactate shuttling. Furthermore, the upregulation of autophagic pathways in CAFs contributes to the recycling of intracellular components, fueling the production of metabolites that sustain tumor cells [13]. The implications of the reverse Warburg effect extend beyond energy metabolism. It has been linked to enhanced resistance to therapy and increased metastatic potential in various tumor types [14].

Additionally, the tumor microenvironment plays a critical role in shaping cancer biology. Factors such as hypoxia, nutrient availability, and immune cell infiltration can significantly influence tumor metabolism and overall tumor behavior [15,16].

Among the multiple factors contributing to metabolic plasticity in cancer cells, endoplasmic reticulum stress emerges as a key regulator of metabolic reprogramming. Endoplasmic reticulum stress occurs when protein folding and disposal capacity in the lumen of the reticulum is impaired, leading to activation of the unfolded protein response. In addition to regulating protein quality, the UPR directly modulates cellular metabolism to adapt the cell to adverse conditions. Specifically, endoplasmic reticulum stress induces a number of key metabolic changes, such as increasing aerobic glycolysis, reducing reliance on oxidative phosphorylation, and promoting the production of metabolic intermediates for biosynthesis. The remodeling of lipid metabolism, with increased phospholipid synthesis to support reticulum expansion and lipid accumulation, may contribute to lipotoxicity and inflammation. Reorganization of amino acid metabolism, with particular emphasis on reliance on glutamine to support nucleotide biosynthesis and glutathione production, is crucial for redox defense. Alterations in nucleotide metabolism related to the increased demand for RNA and DNA synthesis to support high proliferation.

These metabolic adaptations are closely linked to cancer cell survival, contributing to their ability to withstand hostile environmental conditions and evade immune surveillance [17,18,19,20,21,22].

Thus, considering how metabolic alterations and oncogenic metabolites play a crucial role in cancer progression, tumor metabolism is a promising avenue for research into new therapeutic approaches. Targeting specific metabolic pathways or key metabolites could enable the development of targeted treatments capable of interfering with cancer cell growth and survival, thereby improving the efficacy of anticancer therapies. For this reason, this review aims to analyze the major altered pathways in cancer, focusing on how ER stress intersects with these pathways. Additionally, the role of metabolomics is discussed as a powerful tool for identifying potential biomarkers and therapeutic targets that could be leveraged to improve cancer treatment outcomes.

### 1.1. Metabolic Reprogramming in Cancer: A Focus on Metabolomics and Metabolites

Recent progress in molecular biology and metabolomics has significantly enhanced our understanding of cancer’s underlying mechanisms. In particular, metabolomics, or the comprehensive analysis of small molecules and metabolites (<1500 Da) within biological systems, provides critical insights into the metabolic alterations that occur in cancer cells. Cancer offers an ideal context for metabolomics studies, as biopsies of tumor tissue and biofluids from patients are routinely collected, often alongside samples of adjacent non-cancerous tissue. To date, many and various biofluids have been utilized in metabolomics investigations to identify potential cancer biomarkers [23], including serum [24,25,26,27,28,29], plasma [30], saliva [31], urine [32,33,34], and breath [35,36].

Metabolites are intermediates or end products of the metabolism. Metabolic profiling, or the identification, quantification, and interpretation of the downstream products of multiple protein, genetic, and environmental interactions, reflects a quantitative phenotype of a cell, tissue, or organism [37,38]. Although metabolomics has a wide range of applications, including health and different diseases, drug discovery, pharmacology, toxicology, environment, plants, food, and nutrition, the main efforts focus on prevention, early diagnosis, and management of human health and diseases [38,39,40,41,42,43]. Indeed, by investigating the intricate metabolic pathways altered in cancer, metabolomics has paved the way for identifying specific metabolites that may serve as potential biomarkers for early detection and prognosis.

Unlike other “omics” sciences such as genomics and transcriptomics, metabolomics exhibits enormous chemical complexity. Indeed, while the genome is based on four different bases, their numerous possible combinations, alternative splicing, and the degeneracy of the genetic code contribute to vast transcriptomic diversity. Similarly, proteomics, built from 20 amino acids, is further expanded by post-translational modifications. However, the metabolome surpasses both in sheer chemical diversity, encompassing between 1000 and 200,000 distinct compounds [44]. Given the broad chemical diversity in metabolic profiling, high-field nuclear magnetic resonance (NMR) spectroscopy and high-resolution mass spectrometry (HRMS) are the two most common techniques used in metabolomics to analyze different tissues, biofluids, and cell cultures. The release in 2007 of the Human Metabolome Database (HMDB) (https://hmdb.ca, accessed on 5 January 2025) [45], the world’s largest and most complete organism-specific metabolomic, freely available database, has prompted the research in the field of metabolomics using NMR and HRMS platforms.

Metabolites play a crucial role in various biological processes, including cancer metabolism, where they influence tumor growth, progression, and response to therapy. In the following sections, we will explore the various aspects of metabolic reprogramming in cancer, delving into the specific biomolecules involved. Special attention will be given to the most relevant metabolites associated with these alterations and their impact on tumor biology, growth, and therapeutic responses.

### 1.2. Glucose and Main Amino Acids

#### 1.2.1. Glucose

**Figure 1:**
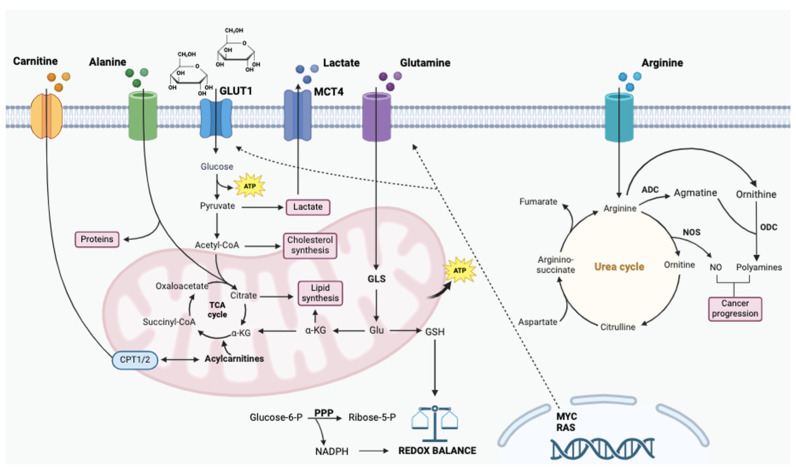
Schematic representation of the main metabolic pathways involved in cancer. The Warburg effect describes the preference for aerobic glycolysis over oxidative phosphorylation, even in the presence of oxygen. In tumors, the upregulation of glucose transporters, such as GLUT1, promotes glucose uptake, which fuels glycolysis: it converts glucose into pyruvate, which is subsequently transformed into acetyl-CoA, feeding the TCA cycle. This process generates ATP and key intermediates such as citrate and α-ketoglutarate (α-KG), essential for fatty acid synthesis. Glucose is also directed into the pentose phosphate pathway (PPP), generating ribose-5-phosphate and NADPH, necessary for redox balance. The generated pyruvate is reduced to lactate by LDH, and the excess lactate produced by cells is exported into the extracellular space via monocarboxylate transporters MCT4 (monocarboxylate transporter 4). Glutamine participates in glutaminolysis, a process that converts glutamine to glutamate (Glu), via glutaminase (GLS), and then to α-ketoglutarate, fueling the TCA cycle. Glutamate is a precursor in the synthesis of glutathione (GSH), which is a potent antioxidant. In cancer, MYC and RAS promote glucose uptake and regulate glutamine metabolism. In the tumor microenvironment, alanine metabolism contributes to protein biosynthesis and acts as an anaplerotic substrate to replenish TCA cycle intermediates. Arginine is converted to nitric oxide (NO) via nitric oxide synthesis (NOS). ADC (arginine decarboxylase) catalyzes the decarboxylation of arginine, converting it to agmatine. Arginine enters the urea cycle and is converted to ornithine. Ornithine, via ornithine decarboxylase (ODC), forms polyamines, which, together with NO, are involved in tumor growth. Carnitine may help cancer cells adapt to low-oxygen conditions by providing an alternative energy source that is less reliant on glycolysis. Created with BioRender.com.

Glucose is crucial to glycolysis, resulting in the conversion of glucose to pyruvate, which is subsequently converted to acetyl-CoA, feeding the TCA cycle (Figure 1). This process not only generates ATP through substrate-level phosphorylation but also produces key intermediates such as citrate and α-ketoglutarate, which are pivotal for the synthesis of nucleotides and fatty acids [46]. Furthermore, the diversion of glucose into the pentose phosphate pathway (PPP) facilitates the generation of ribose-5-phosphate and NADPH, essential for anabolic reactions and redox balance [47]. The upregulation of glucose transporters, particularly GLUT1, enhances glucose uptake in many cancer types, allowing tumor cells to meet their elevated metabolic demands [48]. This metabolic reprogramming is often accompanied by alterations in key regulatory enzymes such as hexokinase and phosphofructokinase, which facilitate the glycolytic flux (Figure 1).

The Warburg effect is commonly observed in cancer cells, characterized by a preference for aerobic glycolysis over oxidative phosphorylation, even in the presence of adequate oxygen levels. This shift enables cancer cells to rapidly generate ATP while producing metabolic intermediates essential for anabolic processes. On the one hand, the Warburg hypothesis highlights the role of aerobic glycolysis in cancer cells. On the other hand, research has established that hypoxia is a common characteristic of these cells [49]. Additionally, it has been observed that mitochondrial dysfunction is not a universal phenomenon in cancer cells [50,51]. The discussions concerning the Warburg effect were resolved with the introduction of its revised version in 2009 [52]. In this model, CAFs release many factors into the tumor microenvironment to induce oxidative stress in adjacent stromal cells such as hydrogen peroxide. The oxidative stress induced by cancer cells damages stromal cell mitochondria, impairing oxidative phosphorylation and forcing these cells to rely on glycolysis as their primary energy source [53]. Glycolysis produces ATP less efficiently than mitochondrial respiration, but it ensures energy production under conditions of mitochondrial dysfunction. To sustain glycolysis, the pyruvate generated is reduced to lactate by LDH, a process that regenerates NAD⁺ from NADH, maintaining the continuity of glycolytic flux [54]. Excess lactate produced by stromal cells is exported into the extracellular space via monocarboxylate transporters MCT4 (monocarboxylate transporter 4) [55]. This leads to the activation of CAFs, which, then, engage in aerobic glycolysis, resulting in the production of high levels of energy-rich metabolites such as pyruvate and ketone bodies [56]. These metabolites are utilized in the mitochondrial TCA cycle and oxidative phosphorylation (OXPHOS) in cancer cells, generating substantial amounts of ATP. Consequently, tumor cells exhibit enhanced proliferative capacity, establishing a metabolic interplay between tumor and stromal cells. This interaction allows tumor cells to better adapt to fluctuations in oxygen availability, transitioning between glycolysis and oxidative phosphorylation to ensure survival. Beyond energy production, oxidative stress in stromal cells activates glycolysis to support antioxidant defense mechanisms. The production of reduced glutathione (GSH), a critical cellular antioxidant, requires NADPH, which is predominantly generated through the PPP [57]. The PPP depends on glucose-6-phosphate, linking its activity directly to glycolysis. Tumor cells demonstrate the Warburg effect under hypoxic conditions and the “reverse Warburg effect” under normoxic conditions, both of which contribute to tumor growth and metastasis [56,58,59]. A similar transition to a glycolytic metabolic profile is evident in immune cells present in the tumor microenvironment, resulting in competition for nutrients between cancer cells and tumor-infiltrating immune cells.

Recent investigations have demonstrated that targeting the upregulated glycolysis in cancer cells is a promising approach to enhancing their susceptibility to various conventional therapies, including chemotherapy, radiotherapy, hormonal therapy, immunotherapy, and photodynamic therapy [60].

#### 1.2.2. Glutamine

Glutamine is also a central player in cellular metabolism, fulfilling crucial roles as a nitrogen donor and carbon source for biosynthetic and energy-producing pathways (Figure 1). In cancer, its significance is heightened by the phenomenon of “glutamine addiction”, where tumor cells exhibit an increased reliance on glutamine to sustain their growth, survival, and adaptability under metabolic stress [61]. Among its key metabolic roles, glutamine takes part in glutaminolysis, a process that converts glutamine into glutamate and subsequently α-ketoglutarate (α-KG), fueling the TCA cycle and supporting anabolic growth [62]. The enzyme glutaminase (GLS), which catalyzes the first step of glutaminolysis by hydrolyzing glutamine to glutamate, has emerged as a critical regulator of cancer metabolism. GLS activity is frequently upregulated in tumor cells, enabling enhanced glutamine utilization to drive energy production, macromolecule biosynthesis, and redox homeostasis [63,64]. This metabolic reprogramming supports tumor cell proliferation and confers a selective growth advantage, particularly in nutrient-deprived or hypoxic microenvironments [65]. Beyond its role in central metabolism, glutaminolysis also contributes to the synthesis of glutathione, a key antioxidant, and other essential metabolites, making it a cornerstone of redox regulation and stress resistance in cancer cells [66].

In the hierarchy of metabolic pathways altered in cancer, the metabolism of glucose and glutamine are frequently reprogrammed due to mutations in key oncogenes and tumor suppressor genes, including Myc proto-oncogene protein (MYC), tumor protein 53 (TP53), RAS-related oncogenes, and signaling pathways such as Liver Kinase B1 (LKB1), AMP-activated protein kinase (AMPK), and phosphoinositide 3-kinase (PI3K). Oncogenic Ras promotes glucose uptake by increasing the expression of GLUT1 and enhances the utilization of glucose through anabolic pathways [67,68].

Additionally, RAS plays a critical role in regulating glutamine metabolism, specifically channeling glutamine-derived carbon into pathways that facilitate biosynthesis, maintain redox homeostasis, and ultimately support cell survival and growth [69,70,71] (Figure 1). Elevated levels of MYC and RAS and reduced levels of tumor suppressors such as TP53 [72] induce various metabolic changes by altering gene expression patterns, among which is possible to recognize enhanced glycolysis [71,73], enhanced in part by transcriptional activation of lactate dehydrogenase A (LDHA) [74], and increased mitochondrial biogenesis [75,76]. The convergence of several pathways in glucose and glutamine metabolism probably reflects their abundance as nutrients, as both are essential metabolic factors [77,78]. Metabolic flux analysis, utilizing techniques such as NMR or mass spectrometry, provides a robust method for mapping altered metabolic pathways in cancer cells. This is achieved by tracing the incorporation of ^13^C-labeled metabolites derived from treatments with ^13^C-glucose or ^13^C-glutamine [79].

#### 1.2.3. Alanine

Alanine is a non-essential amino acid that fuels multiple metabolic pathways important for cancer cell growth, including the TCA cycle, non-essential amino acid synthesis, and nucleotide production [80] (Figure 1). Beyond its classical role in the glucose–alanine cycle, where it serves as a nitrogen carrier and a gluconeogenic substrate in the liver alanine plays a significant role in the metabolic reprogramming of cancer cells and their surrounding microenvironment. In the tumor microenvironment, alanine metabolism contributes to multiple pathways that support tumor growth and survival. As a substrate for gluconeogenesis, alanine secreted by stromal cells, such as CAFs, can be utilized to generate glucose or lactate, feeding the metabolic needs of cancer cells [81]. Additionally, alanine acts as an anaplerotic substrate, replenishing TCA cycle intermediates and sustaining bioenergetics and biosynthesis in cancer cells. Moreover, alanine is intricately connected to other key pathways in the tumor microenvironment, such as lipid biosynthesis and nitrogen metabolism. For instance, alanine-derived pyruvate can contribute to the synthesis of acetyl-CoA, which serves as a precursor for fatty acid biosynthesis, acting as an essential process for rapidly dividing tumor cells [82]. Alanine also intersects with the urea cycle and glutamine metabolism, linking nitrogen transfer and detoxification to the production of biosynthetic precursors. In hypoxic regions of tumors, alanine metabolism is further adapted to support survival and growth. Hypoxia-induced changes in alanine transaminase activity and alanine transporter expression suggest a critical role for this amino acid in maintaining redox balance and energy production under oxygen-limited conditions [83].

MYC-expressing liver cancer cells also utilize alanine to promote their proliferation and survival. To investigate the metabolic pathways fueled by alanine in these cells, fully labeled ^13^C-^15^N-alanine was employed as a tracer. The analysis revealed that nearly all intermediates of the TCA cycle, including malate, fumarate, succinate, α-ketoglutarate, and citrate, were labeled with carbon derived from alanine. Furthermore, a significant role for alanine was observed in supporting the synthesis of various classes of nucleotides [80] (Figure 1).

According to the study by Seth J. Parker et al., pancreatic ductal adenocarcinoma (PDAC) cells, through the increased differential expression of the alanine transporter (SLC38A2), can form so-called metabolic niches. In this case, cancer cells “hijack” specific transporters to enhance the transport of nutrients needed to meet their growing metabolic demands for specific substrates [84]. Alanine has been also identified as a marker in several studies, showing increased levels in gastric cancer [85] and leptomeningeal carcinomatosis [86], while a reduction in its levels was noted in ovarian cancer [87,88].

#### 1.2.4. Arginine

Therapeutic strategies targeting arginine metabolism have emerged as promising approaches in cancer treatment. L-arginine, a semi-essential amino acid or “conditionally essential”, is a pivotal molecule in human physiology due to its involvement in numerous metabolic pathways (Figure 1). It serves as a precursor for various bioactive compounds, including nitric oxide (NO), polyamines, creatine, and agmatine, each of which contributes to critical cellular and systemic functions. Arginine serves as a direct precursor for NO, ornithine, and agmatine through three enzymatic reactions. Specifically, arginine is converted into NO and citrulline by nitric oxide synthase (NOS), into ornithine and urea by arginase, and into agmatine by arginine decarboxylase (ADC) [89]. Polyamines—putrescine, spermidine, and spermine—are synthesized from arginine via ornithine and play essential roles in tumor progression. These polycations stabilize DNA, RNA, and chromatin structures, regulate gene expression, and modulate oxidative stress [90]. Elevated polyamine levels are a hallmark of many cancers, driven by overexpression of ornithine decarboxylase (ODC), the enzyme catalyzing the conversion of ornithine to putrescine. Cancer cells often exploit polyamine metabolism to sustain rapid proliferation. For instance, spermidine and spermine regulate autophagy, a process critical for cellular homeostasis and survival under stress conditions like hypoxia and nutrient deprivation in the tumor microenvironment [91]. Agmatine, formed through decarboxylation of arginine by ADC, has garnered attention for its potential tumor-suppressive effects. Unlike polyamines, which promote tumor growth, agmatine has been shown to inhibit cancer cell proliferation by reducing ODC activity and polyamine synthesis [92]. Furthermore, agmatine modulates ion channel activity and interacts with cellular signaling pathways to induce apoptosis and inhibit angiogenesis, suggesting therapeutic potential in oncology.

This designation arises from the fact that normal cells can synthesize it from citrulline and aspartate through the action of the enzymes argininosuccinate synthetase 1 (ASS1) and argininosuccinate lyase (ASL) in the urea cycle [93]. Several studies have demonstrated that tumor cells with low or absent expression of ASS1 are auxotrophic for arginine, making them vulnerable to therapies that reduce the availability of this amino acid [94,95]. However, under conditions of low arginine availability, immune suppression can occur due to arginine’s critical role in T-cell function, including activation, proliferation, and cytokine production. In fact, low levels of arginine can inhibit the function of T cells and natural killer (NK) cells, which are key players in immune surveillance against tumors. In this regard, arginine replacement therapy has been explored in immune-oncology contexts to restore T-cell activity and enhance immune responses [96].

Despite the relatively straightforward biochemistry of NO, its involvement in tumor biology is highly complex. Research indicates that NO can exert contradictory effects, influencing tumor initiation, promotion, and progression in opposing ways.

Chronic inflammation has long been associated with malignancy and carcinogenesis in various organs, and prolonged exposure to NO induced by chronic inflammation can promote cancer development. Specifically, NO can promote angiogenesis, the formation of new blood vessels, which provides tumors with the nutrients and oxygen they need to grow and metastasize [97]. Furthermore, NO can induce genomic instability by interfering with DNA repair mechanisms and promoting mutations that fuel cancer cell proliferation. It can also enhance the recruitment of inflammatory cells to the tumor site, further promoting a cycle of inflammation that supports cancer progression [98]. Additionally, NO can dampen the body’s immune response to tumors by affecting T-cell function. Elevated levels of NO suppress T-cell activation and the production of antitumor cytokines, leading to immune evasion by the tumor [99]. However, in some cases, the inhibition of NOS has stimulated the synthesis of polyamines. For instance, the NOS inhibitor L-NAME promoted preneoplastic changes induced by carcinogens in a rat colon carcinogenesis model by inhibiting NOS activity and stimulating polyamine synthesis [100,101,102,103]. The overall biological impact of NO production depends on multiple factors, including its concentration, temporal dynamics, the originating cell type, and the characteristics of the target cells [103] (Figure 1).

#### 1.2.5. Carnitine

Carnitine is a quaternary amine, which plays a crucial role in cellular metabolism primarily by facilitating the transport of long-chain fatty acids into the mitochondria for β-oxidation (Figure 1). In the context of cancer, altered energy metabolism is a hallmark feature, and carnitine has emerged as a significant modulator of this metabolic shift. In many cancers, including breast, prostate, and colorectal cancer, elevated levels of carnitine have been observed, correlating with enhanced fatty acid oxidation and increased mitochondrial function, both of which support the energy demands of growing tumors [104] (Figure 1). Carnitine, by supporting mitochondrial fatty acid oxidation, can help tumor cells adapt to low-oxygen conditions by providing an alternative energy source that is less reliant on glycolysis. This adaptation is particularly important for tumor progression and resistance to therapies targeting glycolytic pathways [105].

A study by Fong et al. [106] reported elevated concentrations of carnitine, acetylcarnitine, butyrylcarnitine, and propionylcarnitine, fatty acid transporters, in individuals with both ovarian cancer (OC) and metastatic OC [107].

Interestingly, carnitine has also been suggested to play a role in modulating the immune response within the TME. Studies have shown that carnitine supplementation can influence the polarization of macrophages and affect the inflammatory landscape of tumors, potentially influencing tumor progression and response to treatment [108].

#### 1.2.6. Lipids

##### Fatty Acids

In cancer cells, the primary carbon source for fatty acid synthesis is glucose. Glucose-derived carbon is converted into acetyl-CoA, which then forms citrate in the mitochondria. The mitochondrial citrate transporter (CTP) facilitates the transfer of citrate excess from the mitochondria to the cytosol [109,110]. ATP citrate lyase (ACLY), a key enzyme in de novo lipid synthesis, cleaves cytosolic citrate into acetyl-CoA and oxaloacetate. Cytosolic acetyl-CoA is then utilized for fatty acid biosynthesis [109].

Endogenous fatty acid synthesis is often upregulated in cancer, as fatty acids can be utilized as substrates to produce lipid signaling molecules, modify protein functions through lipidation, synthesize phospholipids for cell membranes, or store energy as triglycerides. Research shows that the expression of metabolic enzymes involved in this synthesis is significantly increased in various types of cancer, such as ACLY in hepatocarcinoma (HCC) [111], acetyl-CoA carboxylase (ACC) in non-small cell lung cancer (NSCLC) [112], fatty acid synthase (FASN) in breast cancer [113], stearoyl-CoA desaturase-1 (SCD1) in ovarian cancer [114], fatty acids desaturase (FADS) 1 in pancreatic cancer [115], FADS2 in HCC and NSCLC [116], FA elongase 2 (ELOVL2) in renal cell carcinoma (RCC) [117], ELOVL5 in colon cancer [118], ELOVL 6 in HCC [119], ELOVL 7 in prostate cancer [120], carnitine palmitoyl transferase (CPT) 1A in breast cancer [121], and CPT1C in HCC [122,123].

**Figure 2:**
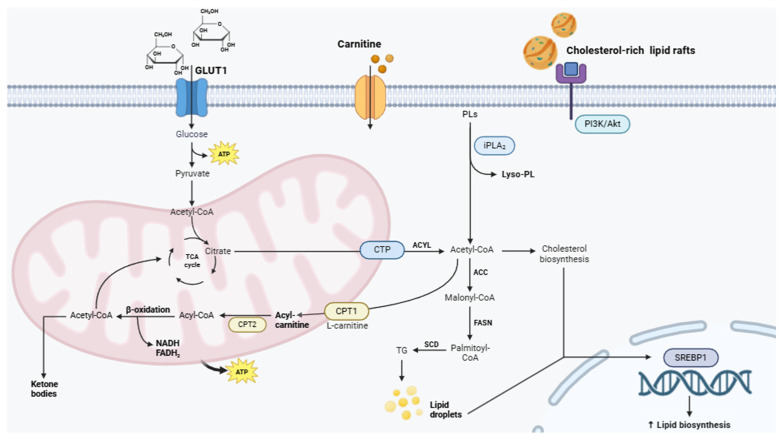
Schematic representation of the main metabolic pathways involved in cancer. In tumors, elevated carnitine is associated with increased fatty acid oxidation and increased mitochondrial function. In cancer cells, glucose is the major carbon source for fatty acid synthesis: glucose is converted to acetyl-CoA and then to citrate in the mitochondria. The enzyme ACLY cleaves citrate to produce acetyl-CoA, which is used for the biosynthesis of fatty acids and cholesterol. Alterations in phospholipid (lysoPL) compositions, triggered by iPLA_2_ (calcium-independent phospholipase A_2_), are linked to tumor progression. Cholesterol-rich lipid rafts in cancer cell membranes act as platforms for receptor clustering and signal transduction, enhancing pathways such as PI3K/protein kinase B (Akt). Cholesterol and lipid accumulation promotes overexpression of SREBP1, enhancing lipogenesis and metastatic potential. Ketone bodies are metabolic by-products of fatty acid oxidation, used by tumor cells as an energy source to fuel rapid proliferation. Created with BioRender.com.

All these alterations indicate a disruption in mitochondrial fatty acid β-oxidation, which has been identified as a potential target for anticancer therapies [124] (Figure 2).

##### Phospholipids

In cancer cells, the primary carbon source for fatty acid synthesis is glucose. Glucose-derived carbon is converted into acetyl-CoA, which then forms citrate in the mitochondria. The mitochondrial CTP facilitates the transfer of citrate excess from the mitochondria to the cytosol [110]. ACLY, a key enzyme in de novo lipid synthesis, cleaves cytosolic citrate into acetyl-CoA and oxaloacetate. Cytosolic acetyl-CoA is then utilized for fatty acid biosynthesis [110].

The phospholipid composition showed notable alterations in the serum of patients with breast [125], colorectal [126], and ovarian cancer [127,128]. The levels of phosphatidylglycerol (PG), ceramide (Cer), sphingomyelin (SM), phosphatidylethanolamine (PE), and lyso-phosphatidylcholine (lysoPC) show notable differences in the plasma of colorectal cancer patients compared to healthy individuals (Figure 2).

PG, a glycerophospholipid, is involved in maintaining mitochondrial function and cellular signaling. Altered PG metabolism in cancer has been linked to enhanced mitochondrial activity and metabolic reprogramming, which supports the high energy demands of rapidly proliferating tumor cells [129].

Similarly, Cer has been shown to act as a signaling molecule that regulates apoptosis, cell cycle progression, and autophagy. In colorectal cancer, an increased concentration of Cer in the plasma is associated with the promotion of tumor cell survival and resistance to apoptosis [130].

Elevated SM levels in CRC patients have been linked to enhanced cell proliferation and migration, which are critical steps in cancer metastasis [131].

PE has been found to be involved in the regulation of cell division and autophagy. Changes in PE levels in CRC patients can reflect alterations in cellular homeostasis and metabolic processes that facilitate tumor progression [132].

Lastly, lysoPC is associated with increased inflammation and tumor progression. Elevated levels of lysoPC in the plasma of colorectal cancer patients have been linked to enhanced inflammatory responses and tumor cell migration, contributing to the metastatic potential of CRC [133].

These variations indicate that these compounds could be useful biomarkers for detecting cancer [134]. Even, in patients diagnosed with OC, there are frequent alterations in free fatty acids and metabolites associated with FA oxidation.

##### Sterols

Sterols are crucial lipid molecules known for their structural role in cellular membranes and their function as precursors for bioactive compounds such as steroid hormones, bile acids, and vitamin D. They are integral to maintaining membrane stability, fluidity, and signaling processes within cells [135]. In cancer, sterol dysregulation influences various cellular processes, including membrane dynamics, energy metabolism, and intracellular signaling pathways. These changes enable cancer cells to sustain rapid growth, evade cell death mechanisms, and adapt to hostile environments [136,137]. For instance, cholesterol-rich lipid rafts in cancer cell membranes act as platforms for receptor clustering and signal transduction, enhancing pathways like PI3K/protein kinase B (Akt) and mitogen-activated protein kinase (MAPK), which promote proliferation and survival [138,139]. Two recent studies have demonstrated that basal-like and human epidermal growth factor receptor 2 positive (HER2+) breast cancer (BC) cells exhibit elevated de novo lipogenesis (DNL) rates when proliferating in brain metastatic sites compared to their primary tumors. This metabolic reprogramming, marked by increased synthesis of cholesterol species and structural lipids, supports the ability of these cells to adapt and thrive in the lipid-rich microenvironment of the brain. The DNL signature, characterized by an increase in cholesterol species and structural lipids, plays a critical role in their capacity to metastasize specifically to the brain. These findings align with clinical observations indicating an upregulation of sterol regulatory element-binding transcription factor 1 (SREBF1) and FASN in brain metastases compared to extracranial metastases or matched primary tumors in patients with metastatic BC [140,141,142]. For example, silencing FASN using siRNA or pharmacological inhibitors, such as orlistat, reduced cell viability and suppressed lipid synthesis in BC cells cultured under brain-derived growth factor-enriched conditions [143]. Additionally, overexpression of SREBP1 has been shown to enhance the metastatic potential of BC cells in brain organoid models, further emphasizing the role of DNL in brain metastasis [144] (Figure 2).

##### Ketone Bodies

Ketone bodies, primarily β-hydroxybutyrate (BHB), acetoacetate (AcAc), and acetone, are metabolic byproducts of fatty acid oxidation produced in the liver under conditions of fasting, caloric restriction, or low carbohydrate availability. Recent evidence suggests that ketone bodies are significantly altered in the TME, contributing to tumor metabolism and progression. Tumor cells, which often exhibit metabolic plasticity, can utilize ketone bodies as an energy source to fuel rapid proliferation (Figure 2). For instance, AcAc has been shown to support oxidative phosphorylation in some cancers, enhancing their growth and survival in nutrient-deprived environments [145]. Additionally, ketone body metabolism is associated with redox balance in cancer cells, as BHB can influence the NAD+/NADH ratio, promoting resistance to oxidative stress [146]. The altered metabolism of ketone bodies in the TME may also have immunomodulatory effects. BHB has been shown to suppress pro-inflammatory pathways in macrophages and other immune cells, potentially contributing to an immunosuppressive TME that favors tumor progression [147]. Moreover, CAFs in the TME may produce and secrete ketone bodies, which are subsequently metabolized by tumor cells, highlighting the cooperative metabolic interplay between stromal and cancer cells [148].

#### 1.2.7. Nucleic Acids and Folates

##### DNA and RNA

DNA, RNA, and their derivatives play multifaceted roles in the TME, influencing cancer progression, immune modulation, and therapeutic resistance. Tumor-derived nucleic acids can be released into the TME through various mechanisms, such as cell death, exosomal secretion, or active extrusion, and they serve as critical mediators of communication between cancer cells and surrounding stromal and immune cells. Extracellular DNA (exDNA) is often detected in the TME, particularly in regions of high necrosis or apoptosis. exDNA contributes to the formation of neutrophil extracellular traps (NETs), which can promote tumor cell adhesion, migration, and metastasis [149]. Moreover, exDNA serves as a ligand for pattern recognition receptors (PRRs) such as Toll-like receptor 9 (TLR9), activating downstream signaling pathways that can support cancer progression by inducing pro-inflammatory cytokines and enhancing tumor-promoting inflammation [150].

Similarly, extracellular RNA (exRNA) plays a vital role in the TME. Tumor-derived exRNAs, including microRNAs (miRNAs) and long non-coding RNAs (lncRNAs), are often packaged into extracellular vesicles like exosomes and transported to nearby or distant cells. These exRNAs regulate gene expression in recipient cells, influencing processes such as angiogenesis, immune evasion, and metastasis [151]. For example, miR-21, a well-studied oncomiRNA, is frequently elevated in the TME and contributes to immune suppression by targeting PTEN and promoting the expansion of myeloid-derived suppressor cells (MDSCs) [152].

**Figure 3:**
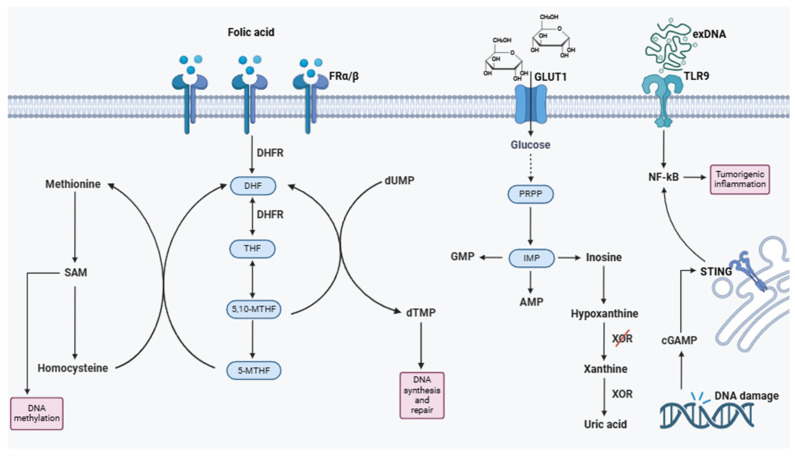
Schematic representation of the main metabolic pathways involved in cancer. The red line indicates the reduction in xanthine oxidase (XOR) expression, which is correlated with various forms of cancer. Folate, as 5-MTHF helps convert homocysteine to methionine, essential for DNA methylation. However, folate deficiency or excess can disrupt DNA repair and cell replication. Additionally, folate receptors (FR-α and FR-β) are overexpressed in many tumors. In cancer cells, purine nucleotides are synthesized via the de novo biosynthetic pathway: PRPP is generated directly to form IMP, which in turn contributes to the production of various intermediates such as AMP, GMP, and inosine. Hypoxanthine, a key purine derivative, is linked to tumor progression. Loss of xanthine oxidase (XOR) expression is related to various forms of cancer. The exDNA acts as a ligand for Toll-like receptor 9 (TLR9) and activates the NF-kB pathway, enhancing tumor-promoted inflammation. Cyclic GMP-AMP (cGAMP), synthesized in response to DNA damage, activates the STING pathway, supporting tumor inflammation. Created with BioRender.com.

Nucleic acids also influence the immunogenic landscape of the TME. Double-stranded RNA (dsRNA), derived from viral infections or cellular stress, can activate innate immune pathways via RIG-I-like receptors (RLRs), such as RIG-I and MDA5. While these pathways can stimulate antitumor immune responses, chronic activation often leads to immune tolerance, favoring tumor growth [153]. Furthermore, cyclic GMP-AMP (cGAMP), a secondary messenger synthesized in response to DNA damage, activates the STING pathway, driving the production of type I interferons and modulating antitumor immunity. However, excessive cGAMP signaling can paradoxically support tumor progression by promoting chronic inflammation and immunosuppressive myeloid cell recruitment [154] (Figure 3).

##### Purines

In non-tumorigenic mammalian cells, purine nucleotides are synthesized through two distinct pathways: the salvage pathway and the de novo biosynthetic pathway. Typically, the salvage pathway meets most of the cell’s purine requirements by recycling degraded bases with the help of hypoxanthine–guanine phosphoribosyltransferase (HPRT) and adenine phosphoribosyltransferase. HPRT recycles hypoxanthine and guanine by transferring the phosphoribosyl group from phosphoribosyl pyrophosphate (PRPP) to generate inosine monophosphate (IMP) and guanosine monophosphate (GMP), respectively. Under conditions of higher purine nucleotide demand, such as in dividing cells and tumor cells, the de novo biosynthetic pathway is essential for replenishing the purine pool. In this pathway, PRPP is directly generated to form IMP, which in turn contributes to the production of various intermediates such as AMP, GMP, adenosine, and inosine. Inosine is further converted into hypoxanthine by purine nucleoside phosphorylase (PNP), and xanthine oxidase (XO) catalyzes the oxidation of hypoxanthine to form xanthine [155,156,157].

Inosine promotes the proliferation of tumor cells, and an altered ratio between adenosine and inosine has been observed in many tumor cells, influencing growth, invasiveness, and metastasis. Moreover, purines are involved in regulating immune responses, contributing to tumorigenesis. A mutation or deficiency in the enzyme adenosine deaminase, which degrades purine metabolites, can increase susceptibility to infections and autoimmunity, and its activity is used as a tumor marker.

Hypoxanthine, a natural purine derivative, is involved in adenosine metabolism and nucleic acid formation through the purine salvage pathway. During purine catabolism, hypoxanthine is converted into xanthine, which is subsequently transformed into uric acid by xanthine oxidase (XOR), a process associated with the production of ROS [158,159]. Loss of XOR expression has been shown to correlate with the aggressiveness of breast cancer and poor prognosis. Hypoxanthine is recognized as a biomarker of hypoxia, a condition occurring in tumor cells distant from functional blood vessels (Figure 3).

In a study by Shakartalla et al., hypoxanthine was detected via ^1^H-NMR in the conditioned medium of highly metastatic breast cancer cells, but not in that of low-metastatic cells. Hypoxanthine levels in MDA-MB-231 cells were about three times higher than in MCF-7 cells [160]. It was found that hypoxanthine is directly responsible for inducing epithelial-mesenchymal transition (EMT) by increasing the expression of Snail (Snail family transcriptional repressor 1), a key regulator of EMT. Furthermore, genetic knockdown of PNP significantly reduced the expression of mesenchymal markers, likely due to a decrease in hypoxanthine and/or other metabolites. Additionally, in the same study, hypoxanthine was found to significantly increase the levels of proprotein convertase subtilisin/kexin type 9 (PCSK9) [161], suggesting another mechanism by which hypoxanthine may induce metastasis. PCSK9 is a protein that regulates the levels of low-density lipoprotein receptors (LDLR) and plays a critical role in cholesterol homeostasis. Tumor cells in breast cancer, by overexpressing PCSK9, show a high cholesterol content, a factor that accelerates cancer progression and increases resistance to hormonal therapy.

##### Folic Acid

Folate is an essential water-soluble B vitamin, crucial for cellular metabolism. Natural folate is present in its reduced form, bound to polyglutamate chains that must be oxidized and hydrolyzed for absorption, while folic acid is already in its oxidized form as pteroylmonoglutamate, making it more readily available for the body. Folic acid, found in fortified foods and supplements, is converted into tetrahydrofolate in the liver by the enzyme dihydrofolate reductase (DHFR) [162,163].

Folate, in the form of 5-methyltetrahydrofolate (5-MTHF), along with cobalamin, is essential for the conversion of homocysteine to methionine in the methionine cycle. Methionine is then transformed into S-adenosylmethionine (SAM), an important methyl group donor in numerous biological reactions, including the methylation of DNA and RNA. Insufficient SAM production can reduce the methylation of CpG islands in DNA, affecting gene transcription and altering the expression of tumor suppressor genes and proto-oncogenes. Additionally, folate deficiency can prevent the conversion of deoxyuridine monophosphate (dUMP) to deoxythymidine monophosphate (dTMP), a key component in DNA synthesis and repair. This error may lead to the incorporation of uracil instead of thymine, causing DNA instability, strand breaks, and defective DNA repair [164,165].

However, an excess of folic acid administration can saturate DHFR, preventing its reduction, which could be a carcinogenic mechanism. Both folate deficiency and excessive intake can interfere with cell replication and survival, and reduced enzyme efficiency can alter nutrient metabolism and impact disease risk [166].

Considering that folate plays a crucial role in nucleotide synthesis and as a cofactor in the rate-limiting phase of DNA synthesis, it represents a potential growth factor for tumor cells. As a result, there is an overexpression of receptors responsible for its absorption in rapidly dividing cells, such as those in solid tumors. There are two main folate receptors: the α receptor (FR-α) and the β receptor (FR-β), both of which are expressed in malignant tissues. In fact, increased expression of these receptors has been observed in various types of tumors, including ovarian cancer, lung cancer, breast cancer, endometrial tumors, kidney cancer, colorectal carcinoma, myeloid hematopoietic tumors, brain tumors, and placental tumors (Figure 3). Folate receptor expression levels in tumors have also been directly associated with the stage of the disease [167]. Therefore, the folate receptor could represent a useful target to improve the outcomes of cancer therapies. Stanisławska-Sachadyn et al. reported that folate levels higher than the median value (>17.5 nmol/L in healthy individuals) were associated with an increased risk of developing lung cancer among smokers [168]. The link between folic acid and prostate cancer has also been extensively studied. In the research conducted by Rycyna et al., high blood folate levels were found to potentially contribute to the development of prostate cancer [169]. This finding was also confirmed by a meta-analysis, which supported the evidence, as well as by the results of the Aspirin/Folate Polyp Prevention Study (AFPPS), which followed patients with folic acid supplementation for 10 years.

In Table 1, the main metabolites just described are reported, associated with the metabolic alterations involved in tumor growth.

## 2. ER Stress and Unfolded Protein Response Activation

The ER functions as the principal biosynthetic organelle within the cell, orchestrating the synthesis of proteins and lipids [170,171]. The ER is categorized into two distinct forms based on ribosomal presence: smooth ER, which is ribosome-free and primarily involved in lipid biosynthesis, and rough ER, which is studded with ribosomes and plays a critical role in the synthesis and post-translational modification of proteins. The rough ER is pivotal in the assembly and maturation of proteins; it is indispensable for the biosynthesis and modification of proteins destined for organelles like lysosomes, the plasma membrane, or secretion outside the cell. Under physiological conditions, the ER quality control mechanisms rigorously monitor protein biogenesis and maturation to ensure proper conformational folding [172]. Misfolded proteins are identified by molecular chaperones and retrotranslocated into the cytosol via a pathway mediated by Hrd1-mediated autoubiquitination [173]. These aberrant proteins are subsequently directed to the endoplasmic reticulum-associated degradation (ERAD) pathway for ubiquitin–proteasome-mediated degradation [174]. In response to pathological stimuli, such as glycosylation inhibition, disruption of disulfide bonds, imbalance of intracellular ROS, pH fluctuations, and accumulation of damaged DNA, the ER homeostatic equilibrium is perturbed [175,176]. This disruption triggers a cascade of compensatory response pathways aimed at maintaining cellular integrity under ER stress conditions, known as UPR, a mechanism designed to restore ER function [177].

UPR enhances the cell’s capacity to correctly fold proteins, removes misfolded ones more effectively, and regulates overall protein synthesis to manage stress. This response helps to maintain cellular stability under adverse conditions, illustrating how cells adapt to internal stress. UPR is mediated by three key transmembrane sensors within the ER: inositol-requiring enzyme 1 (IRE1), PKR-like endoplasmic reticulum kinase (PERK), and activating transcription factor 6 (ATF6) [177,178,179]. These sensors identify disruptions in ER homeostasis and trigger downstream signaling pathways to mitigate and restore balance. The activation of these sensors and signal transducers is primarily attributed to their dissociation from the chaperone BiP (binding-immunoglobulin protein), also known as the 78-kDa glucose-regulated protein (GRP78). Under physiological conditions, these proteins are inhibited through their binding to the molecular chaperone BiP. Upon activation of the UPR, dissociation of the molecular chaperones from these transducers occurs, initiating three protein-mediated signaling pathways that upregulate the expression of ER stress-related genes. GRP78 is recruited to misfolded proteins to facilitate proper protein folding, and its release from these sensors is a critical step in the activation of the associated signaling pathways [180].

The UPR is a highly specialized monitoring mechanism that detects and responds to ER stress before it threatens the survival of the affected cell. When an abnormal accumulation of proteins is detected, the activated UPR signaling pathways lead to a reduction in protein synthesis, an increased capacity for protein folding, and enhanced removal of misfolded, unprocessed, insoluble, or otherwise damaged proteins, accelerating their degradation.

The UPR response is influenced by the nature, intensity, and duration of the stress. When ER stress becomes irreparable, particularly if severe or prolonged, the UPR may fail and trigger pro-inflammatory and pro-death signals. Cell death is primarily executed by apoptosis, but necrosis, necroptosis, pyroptosis, and autophagy may also contribute. Additionally, the UPR plays a role in regulating cell proliferation, differentiation, and angiogenesis [181,182].

### 2.1. IRE1

In the mammalian genome, two isoforms of IRE1 can be encoded, IRE1α and IRE1β.

IRE1α is ubiquitously expressed, while the expression of IRE1β is restricted to certain tissues.

IRE1α is a type I transmembrane protein characterized by cytosolic serine/threonine kinase and endoribonuclease (RNase) domains, which persistently regulate protein folding capacity, actively guide UPR signaling, and execute cellular fate determination. Both domains of IRE1α are activated through oligomerization of IRE1α, along with the autophosphorylation of its kinase domain. The RNase domain cleaves a 26-nucleotide intron within the unspliced mRNA of X Box Binding Protein 1 (XBP1), known as XBP1u. The resulting RNA fragments are then ligated by RNA 2′,3′-cyclic phosphate and 5′-OH ligase (RtcB), producing the spliced XBP1 mRNA, referred to as XBP1s. XBP1s contains a DNA-binding domain and is a transcription factor that belongs to the basic region/leucine-zipper (bZIP) family. It plays a crucial role in promoting the transcription of UPR-targeted genes that enhance protein folding capacity, such as GRP78 and components involved in ERAD [183]. Activated XBP1s can dimerize with hypoxia-inducible factor 1α (HIF1α) to enhance the expression of hypoxia-sensitive genes, including vascular endothelial growth factor A (VEGF-A), a key mediator of angiogenesis [184]. XBP1s also directly interacts with the proto-oncogene Myc, which drives the expression of IRE1α and the splicing of XBP1 or enhances the transcriptional activity of XBP1.

Additionally, the RNase domain of IRE1α can rapidly degrade a group of mRNAs and microRNAs through a process known as IRE1α-dependent decay (RIDD). This phenomenon seems to be essential for promoting cell survival by limiting the number of redundant peptides entering the ER.

However, once ER stress intensifies, IRE1α ceases to form XBP1s and promotes cell death through RIDD by enhancing the non-specific degradation of mRNAs encoding pro-survival proteins. Furthermore, active IRE1α not only promotes UPR but also mediates other pathways: activated IRE1α interacts with the tumor necrosis factor receptor-associated protein (TRAF-2) to form the IRE1α-TRAF2 complex. This complex interacts with apoptosis signal-regulating kinase 1 (ASK1) to form the IRE1α-TRAF2-ASK1 complex, which associates with receptor-interacting protein kinase 1 (RIPK1), resulting in phosphorylation of c-Jun N-terminal kinase (JNK). The complex also activates IκB kinase (IKK), which phosphorylates IκB, leading to the release of NF-κB and its translocation into the nucleus, where it induces the expression of inflammatory cytokines. Activated JNK can promote the formation of the NLRP3 (NLR family pyrin domain containing 3) inflammasome by phosphorylating the transcription factor AP-1 and induces the expression of caspase-1, IL-1β, IL-18, and other inflammatory genes. IRE1α increases the expression of the protein TXNIP, which can also activate the NLRP3 inflammasome, leading to the dissociation of caspase-1 and the secretion of IL-1β. Activated caspase-1 can also trigger pyroptosis by cleaving gasdermin D to generate an N-terminal fragment that binds to the inner leaflet of the plasma membrane to form pores. JNK activation regulates various members of the Bcl-2 family, particularly pro-apoptotic factors Bcl-2-like protein 11 (BIM) and BH3 interacting domain death agonist (BID). At the same time, JNK triggers downstream autophagy mediators.

**Figure 4:**
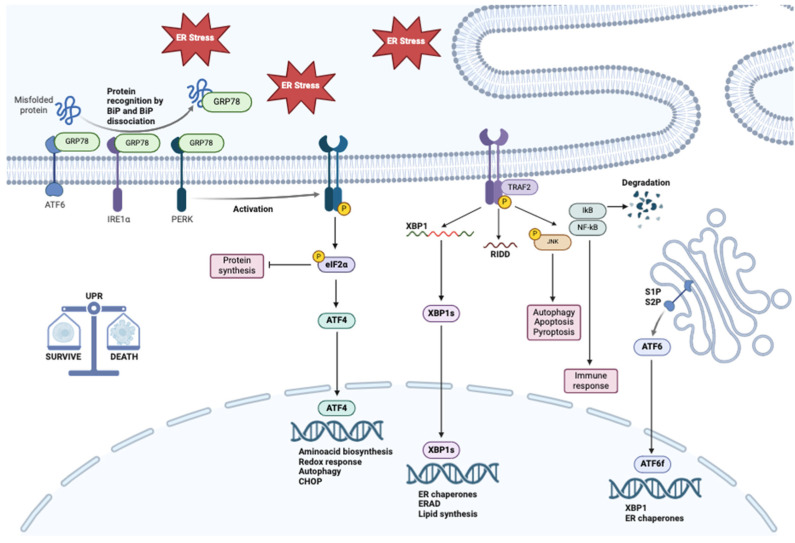
UPR pathway. The red line indicates XBP1s. IRE1 pathway: in response to ER stress, IRE1 undergoes oligomerization and autophosphorylation, triggering the splicing of XBP1. The spliced form of XBP1 (XBP1s) acts as a transcription factor to activate genes associated with the unfolded protein response (UPR). Additionally, the RNase domain of IRE1α mediates the degradation of specific mRNAs and microRNAs through a process called IRE1α-dependent decay (RIDD). IRE1α also interacts with tumor necrosis factor receptor-associated protein (TRAF-2), resulting in the phosphorylation of c-Jun N-terminal kinase (JNK) and in the degradation of IκB, which leads to the activation of NF-κB signaling. PERK pathway: PERK phosphorylates eIF2α, which in turn stimulates ATF4. ATF4 regulates the expression of genes involved in the cellular stress response. ATF6 pathway: ATF6 is cleaved by the proteases S1P and S2P to produce its active form, ATF6f. ATF6f then translocates to the nucleus, where it initiates the transcription of genes involved in the UPR. Created with BioRender.com.

JNK inhibits the association of Bcl-2 with Beclin-1 and positively regulates Beclin-1 expression by phosphorylating c-Jun. Beclin-1 is associated with autophagy, being the downstream regulator of MAPK8, and is activated by direct phosphorylation of Bcl-2, which then disrupts the interaction between Beclin-1 and Bcl-2. This event activates the PI3K complex and induces autophagy in cancer cells. Furthermore, it has been noted that overexpression of XBP1s enhances the conversion of LC3-I to LC3-II and increases Beclin-1 expression [185,186,187,188] (Figure 4).

### 2.2. ATF6

ATF6 is synthesized as a type II transmembrane glycoprotein and is incorporated into the ER membrane. ATF6 is activated in response to ER stress and is subsequently transported to the Golgi apparatus, where it undergoes sequential cleavage by sphingosine-1-phosphate (S1P) and site-2 protease (S2P) proteases, resulting in the generation of the transcriptionally active fragment ATF6f [180,189].

The cytosolic domain of ATF6 is a transcriptional activator for BiP, CHOP, and other chaperones and promotes protein-folding homeostasis. Meanwhile, the cleaved transcription factor domain of ATF6 (ATF6f) enters the nucleus to modulate the transcription of UPR target genes. The ATF6 and IRE1α branches of the UPR signaling pathway are interconnected, as both positively regulate XBP1, involved in BiP synthesis, protein folding, quality control, and ERAD-associated proteins (Figure 4). ATF6 is believed to primarily induce a cytoprotective response, including ER biogenesis, chaperone expression, and protein degradation, although a link has been found between ATF6 and the indirect downregulation of a pro-survival Bcl-2 family member, the myeloid cell leukemia sequence 1 (MCL1).

Other known targets of ATF6 include the α-mannosidase-like protein 1 (EDEM1), which enhances ER degradation, and the disulfide isomerase protein 6 (PDIA6), which promotes the degradation of misfolded proteins.

Furthermore, compelling evidence has demonstrated the role of ATF6α in the development and differentiation of different cell types [190,191,192,193,194,195].

### 2.3. PERK

PERK is a type I transmembrane protein, structurally similar to IRE1α, and is kept in an inactive state due to its association with the chaperone GRP78. When ER stress occurs, PERK dissociates from GRP78 and undergoes oligomerization and trans-autophosphorylation, leading to the phosphorylation of eukaryotic translation initiation factor 2α (eIF2α). This phosphorylation effectively halts the translation of most mRNAs, resulting in a reduction of overall protein synthesis while promoting the production of stress-related proteins. Consequently, the transcription of most proteins is downregulated, leading to decreased accumulation of proteins in the ER lumen and alleviating ER stress. Once phosphorylated, eukaryotic initiation factor 2α (eIF2α), which is a component of eIF2, loses its activity. This phosphorylation has significant physiological, pathological, and therapeutic implications. Notably, specific kinases target eIF2α for phosphorylation, selectively upregulating the translation of activating transcription factor 4 (ATF4) in response to various microenvironmental stresses. ATF4 acts as a transcriptional activator, translocating to the nucleus to positively regulate genes involved in amino acid synthesis, redox homeostasis, protein maturation, and degradation [196]. Finally, ATF4 is the main activator of CHOP (C/EBP homologous protein), a transcription factor that plays a key role in the cellular stress response. Its activation helps mediate effects such as autophagy and cell death, especially under conditions of prolonged stress (Figure 4).

The NF-E2-related factor 2 (Nrf2), a protein belonging to the basic leucine zipper (bZIP) family, has also been described as a substrate of the PERK kinase. Its phosphorylation-dependent activation regulates the cellular antioxidant response, which is necessary to balance the increase in ROS associated with ER stress.

Recent studies have identified a specialized subdomain of the ER, known as the mitochondria-associated membrane (MAM) [197], which facilitates direct communication between the ER and mitochondria. This tethering is thought to play a critical role in lipid and calcium trafficking between the two organelles, thereby regulating lipid metabolism and maintaining mitochondrial calcium homeostasis [197,198,199,200]. Furthermore, the MAM is involved in the de novo synthesis of phospholipids and their transport between the ER and mitochondria. Several proteins associated with the UPR, including ER chaperones, calcium channels, and ER-resident oxidoreductases, have been found to localize to the MAM [201,202,203]. Notably, the protein PERK has been shown to interact directly with mitofusin 2 (MFN2), which forms a molecular bridge between the ER and mitochondria. Deletion of MFN2 leads to ER stress and activates all three branches of the UPR signaling pathways PERK, ATF6, and IRE1α [204].

## 3. The Role of ER Stress in Cancer

Even in tumor cells, ER stress and UPR activation can help restore homeostasis and make the surrounding environment conducive to tumor survival and expansion.

As previously reported, one of the most common characteristics of cancer cells is their ability to metastasize to other tissues, where unfavorable environmental conditions, such as hypoxia, glucose deprivation, lack of growth factors, lactic acidosis, oxidative stress, and amino acid deficiency, threaten the proper folding of proteins in the ER. In addition, various intrinsic stresses shared by many cancer cells, such as oncogene activation, aneuploidy, and increased glycolysis, can lead to an increase in protein synthesis and secretion, placing further pressure on the protein secretion system. Genomic instability and somatic mutations in proteins involved in the secretory pathway can disrupt their proper folding, leading to ER stress. During tumorigenesis, the high proliferation rates of cancer cells require increased activity of protein folding, assembly, and transport within the ER—conditions that can lead to physiological stress [205,206]. For this reason, numerous studies have documented high activation of all three branches of the UPR in a wide range of hematologic and solid tumors, including glioblastoma, and carcinomas of the breast, stomach, colon, esophagus, lungs, prostate, pancreas, and liver [207,208].

Despite extensive evidence of ongoing ER stress in various types of cancer, it remains a topic of debate whether the UPR limits or promotes tumor growth, as the downstream branches of the UPR play divergent roles in different malignancies, acting either as oncogenic drivers or tumor suppressors [178]. This is because, during neoplastic transformation, UPR activation helps to manage ER stress, but must be carefully regulated to avoid cell death: a too-low UPR does not support proliferation, while a too-high UPR induces cell death. Some protective mechanisms put in place by tumor cells include suppression or mutation of transducers or induction of autophagy, which removes damaged proteins and reduces oxidative stress.

Thus, the activation of the UPR is a strategy that tumor cells use to survive, but this must be finely regulated to avoid the activation of death signals, which explains the paradoxical effects caused by its main mediators [183].

ER stress and activation of the UPR signaling pathway have an essential impact on all stages of cancer progression. Indeed, in recent years, numerous preclinical studies have provided strong evidence that the UPR supports various aspects of tumor growth, including angiogenesis, metabolism, metastasis, and chemoresistance. For example, studies have shown that the homeostatic target of IRE1α, XBP1, promotes tumor progression in models of triple-negative breast cancer cells. Genetic deletion of IRE1α in a human glioma cell line inhibited angiogenesis and reduced tumor growth when these cells were subsequently injected into mice [209].

The activation of the PERK, IRE1, and, to a lesser extent, ATF6 signaling pathways has been observed in various cancers following oncogene activation. For instance, the BRAF V600E mutation triggers the activation of both IRE1 and ATF6 in melanoma cells [210]. Additionally, B cells derived from Burkitt’s lymphoma patients, which overexpress c-MYC, display increased phosphorylation of PERK and eIF2α, along with elevated levels of XBP1s and ATF4, compared to B cells from healthy donors [211]. Selective activation of c-MYC in mouse embryonic fibroblasts (MEFs) expressing a tamoxifen-inducible c-MYC (mycER) similarly leads to the activation of both PERK and IRE1. Furthermore, the activation of UPR has also been documented following H-RAS activation in melanocytes and keratinocytes [183,212].

Notably, UPR and autophagy are tightly interconnected, as the UPR activates autophagy. This latter shows an ambiguous role in cancer, acting both as a survival mechanism and as an inhibitor of tumor progression. However, increasing evidence suggests that UPR-induced autophagy is crucial for the survival of tumor cells, particularly under stress conditions [213]. For example, [214] in melanoma, the BRAF inhibitor PLX4720 induces ER stress-mediated autophagy via the PERK kinase. Inhibition of autophagy reduces melanoma resistance to treatment, suggesting that autophagy is essential for tumor cell survival. Similarly, in HCT116 and DU145 cell lines, UPR-induced autophagy promotes survival in response to ER stress inducers [215]. However, in normal cells, suppression of autophagy increases cell death, indicating that while autophagy can protect tumor cells, it may instead induce cell death in healthy cells.

In breast cancer cells, MCF-7 and MDA-MB-231, treated with tunicamycin, excessive UPR activation was modulated by autophagy activation, regulated by IRE1/JNK/beclin-1. Autophagy alleviates ER stress by removing ubiquitinated proteins, thus playing a protective role [216].

Below, we outline the main UPR transducers and their roles in cancer progression and adaptation.

### 3.1. GRP78

GRP78 is recognized as a molecular chaperone within the ER and is upregulated in response to ER stress. Beyond its ER localization, GRP78 is also present in the plasma membrane, cytoplasm, mitochondria, nucleus, and extracellular fluids. It plays a significant role in various aspects of tumor biology, including cell proliferation, resistance to cell death, immune evasion, metastasis, and angiogenesis. As a key sensor of stress, GRP78 is instrumental in detecting and adapting to changes in the tumor microenvironment [217]. The expression of GRP78 is positively associated with greater tumor thickness and a higher mitotic index in the dermal tissue [218]. In various human malignancies, increased levels of GRP78 correlate with higher pathological grades, an elevated risk of recurrence, and decreased survival rates in breast, liver, prostate, colon, and gastric cancers [219].

However, in some prostate cancer models, GRP78 levels have been found to be decreased. GRP78 has a multifaceted role, initially functioning as a tumor suppressor by inducing cell dormancy but later promoting tumor progression and metastasis in advanced stages through pro-survival and pro-metastatic functions. These functions are mediated by signaling pathways such as the PI3K/phosphatase and tensin homolog (PTEN)/protein kinase B (PKB) pathways, which are crucial for these processes. In mice, PKB activation in PTEN-null prostate epithelium was strongly suppressed in a GRP78 knockout model, and a similar suppression of PKB activation was observed in human prostate cancer cells that were transfected with small interfering RNA (siRNA) targeting GRP78.

Other studies have highlighted the interaction between cell migration inducing hyaluronidase 1 (CEMIP), a cell migration inducer protein, and GRP78, which helps tumor cells adapt to hypoxia. In this case, CEMIP positively regulates GRP78 levels, facilitating tumor growth and metastasis. Another study showed that inhibition of GRP78 increases the sensitivity of colorectal cancer cells to chemotherapeutics such as 5-fluorouracil (5-FU) and oxaliplatin, improving the response to treatment and promoting apoptosis of cancer cells.

The suppression of GRP78 in endothelial cells prevented their proliferation, survival, and migration. GRP78 directly regulates the expression of vascular endothelial growth factor, a key driver of endothelial cell proliferation, and acts on the VEGF-2 receptor.

In a study by Cholody et al., it is shown that the reduction of GRP78 levels by HKH40A, a synthetic agent that reduces GRP78 transcription, in various cancer cell lines (colon, liver, pancreas, brain) impairs its function and induces UPR, as evidenced by the activation of IRE1α, ATF6, and PERK. This leads to a series of downstream events, including sustained phosphorylation of eIF2α, increased spliced XBP1 mRNA, and elevated protein levels of ATF4 and CHOP, ultimately resulting in cell death [220].

Several FDA-approved and investigational drugs have been shown to modulate GRP78 expression or function, offering a potential strategy for repurposing existing agents in oncology. While GRP78 has been extensively studied as a target for chemotherapy and small-molecule inhibitors, recent evidence suggests that it also plays a critical role in tumor response to radiotherapy. GRP78 is upregulated in response to ionizing radiation, contributing to radioresistance by promoting DNA damage repair, inhibiting apoptosis, and enhancing tumor cell survival [221]. Thus, combining GRP78-targeting strategies with radiotherapy may improve treatment efficacy and overcome resistance mechanisms. Radiotherapy induces DNA damage and oxidative stress in tumor cells, triggering a complex cellular response that includes activation of UPR and GRP78 overexpression [222]. Elevated GRP78 levels have been associated with resistance to radiation in multiple cancer types, including glioblastoma, prostate, and lung cancer [223]. The mechanisms underlying GRP78-mediated radioresistance involve the inhibition of apoptosis where GRP78 suppresses apoptotic signaling by modulating pro-survival pathways, including PI3K/AKT and NF-κB, preventing radiation-induced cell death [224] and the activation of pro-survival autophagy, where the radiation-induced ER stress activates autophagy via GRP78, providing an alternative survival mechanism for cancer cells [225].

The following are several compounds, approved drugs, and investigational agents that have shown potential in targeting GRP78 directly or indirectly, thereby improving the efficacy of conventional therapies and overcoming resistance mechanisms.

- Honokiol: A natural biphenolic compound derived from *Magnolia* spp., honokiol has been reported to downregulate GRP78, inducing ER stress-mediated apoptosis in breast cancer and glioblastoma models [226].
- Metformin: Widely used for type 2 diabetes, metformin has demonstrated anti-cancer effects by inhibiting GRP78 and sensitizing tumor cells to chemotherapy. In colorectal and ovarian cancer models, metformin downregulates GRP78 and disrupts its interaction with AKT, enhancing drug-induced cytotoxicity [227]. Moreover, metformin enhanced the radiosensitivity of cancer stem cells in vitro and significantly improved the response of experimental tumors to irradiation, suggesting that this anti-diabetic drug could be beneficial in increasing the effectiveness of cancer radiotherapy [228].
- HA15: A novel thiazole benzenesulfonamide compound, HA15 selectively targets GRP78 and induces ER stress-mediated apoptosis in melanoma and pancreatic cancer cells. Preclinical studies have shown promising efficacy in overcoming drug resistance and improving tumor regression [229].
- Itraconazole: An antifungal agent, itraconazole inhibits GRP78 and mTOR signaling, leading to reduced proliferation in prostate and lung cancer models [230].
- Novel strategies, including monoclonal antibodies and small-molecule inhibitors, are being developed to enhance specificity while minimizing off-target effects [231].

### 3.2. IRE1

It has been shown that the homeostatic target of IRE1α, XBP1, promotes tumor progression in models of triple-negative breast cancer cells. Genetic deletion of IRE1α in a human glioma cell line inhibited angiogenesis and reduced tumor growth when these cells were subsequently injected into mice [209]. Specifically, IRE1 promotes cell proliferation by regulating the expression of cyclin A1 in prostate cancer cells, and the splicing of XBP1 increases the expression of catalase, protecting the cells from oxidative stress. An IRE1 RNase inhibitor showed anti-myeloma activity in mouse models, suggesting that the IRE1-XBP1 pathway could be a target for cancer treatments.

Studies have found that mutations in the IRE1α gene disable its antiproliferative effects, suggesting that some cancer cells may deactivate the UPR to survive. Similarly, a loss-of-function mutation in XBP1, which prevents the proper splicing of its mRNA, has been reported in rare cases of myeloma and may be associated with resistance to proteasome inhibitors [232,233].

When IRE1 is activated, it recruits the protein TRAF2, which activates JNK kinase and leads to caspase-12 activation and apoptosis. In a tumor context, the overexpression of XBP1 in breast cancer cells increased levels of Bcl-2, inhibiting apoptosis, but JNK can phosphorylate and inhibit Bcl-2, showing how the effects of IRE1 can vary depending on the output: pro-apoptotic when mediated by JNK and anti-apoptotic when mediated by XBP1 splicing. This balance between pro-death and pro-survival outputs is tightly regulated and allows tumor cells to adapt to varying stress conditions, highlighting the complex role of IRE1 in cancer progression [233].

Radiotherapy induces ER stress by generating ROS and disrupting protein homeostasis in cancer cells. This leads to the activation of the IRE1 pathway, which promotes tumor cell survival through various mechanisms [234].

Various approved and investigational drugs have been identified as modulators of IRE1 signaling, including the following:

- 4μ8C and STF-083010: these small-molecule inhibitors specifically block IRE1’s RNase activity, preventing XBP1 splicing and impairing tumor cell survival. Preclinical studies have shown that 4μ8C enhances the efficacy of chemotherapy in multiple myeloma and TNBC models [235].
- MKC-3946: potent IRE1 inhibitor that sensitizes pancreatic cancer cells to gemcitabine, reducing tumor burden in preclinical models [236].
- Toyocamycin: these natural compounds inhibit IRE1-mediated XBP1 splicing, leading to ER stress accumulation and apoptosis in leukemia and lymphoma cells [237].
- CXC195: a pyrazine compound linked to pro-apoptotic effects in hepatocellular carcinoma cells through the activation of IRE1 and ATF6, along with the suppression of the PI3K/Akt/mTOR signaling pathway [238,239].

### 3.3. ATF6

ATF6 is a key component in the precise regulation of the ER protein response, but its role in cancer has been studied less than the other two transducers. Nevertheless, its potential impact on clinical outcomes should not be overlooked. When activated, the ATF6 transcription factor is more frequently translocated to the nucleus in various types of cancer, such as HCC and Hodgkin’s lymphoma, and has been associated with metastasis and recurrence [240,241]. Recently, ATF6 has been shown to play a crucial role in the survival of quiescent, non-proliferative squamous carcinoma cells and their adaptation to chemotherapy. This process is mediated by the Ras homolog enriched in the brain (RHEB) and the activation of the mammalian target of rapamycin (mTOR) [242]. In fact, inhibiting ATF6 or RHEB was shown to reduce the resistance of dormant cells and prevent recurrence in in vivo models.

Compounds that have emerged as regulators of ATF6 signaling, which could have applications in oncology, include the following:

- Ceapins (Ceapin-A7, Ceapin-272): selective inhibitors that block ATF6 activation by preventing its trafficking to the Golgi. These compounds sensitize breast and prostate cancer cells to chemotherapy by disrupting the ATF6-mediated stress response [243].
- Bortezomib: a proteasome inhibitor approved for multiple myeloma, bortezomib reduces ATF6 signaling by impairing ER stress resolution, leading to increased apoptosis in leukemia and solid tumors [244].
- Hydroxychloroquine (HCQ): an autophagy inhibitor that synergizes with ATF6 depletion, leading to reduced integrin expression and tempering tumor growth and metastasis. Combining HCQ with ATF6 inhibitors may enhance radiosensitivity by disrupting tumor cell survival mechanisms [245].

### 3.4. PERK

Studies have shown that PERK is involved in tumor progression and angiogenesis. Targeted mutagenesis of PERK has reduced tumor growth and impaired angiogenic capacity in mouse models of colon cancer and fibroblasts. Additionally, the loss of PERK in a mouse model of breast cancer increased tumor latency but inhibited metastatic spread [240]. Similarly, in human esophageal and breast carcinomas, the deletion of PERK caused cell cycle arrest at the G2/M phase due to reduced Nrf2 activity and the accumulation of ROS, which subsequently led to DNA damage and activated the cell cycle checkpoint [246]. However, under certain conditions, such as in squamous cell carcinoma, the activation of PERK has been associated with p38-induced cellular dormancy and suppression of tumor growth. This suggests that PERK may have a complex, context-dependent role in regulating tumor growth and spread. Specifically, pharmacological activation of PERK with subsequent phosphorylation of eIF2α can induce tumor growth arrest both in vitro and in vivo, primarily through the inhibition of cyclin D1 and cell cycle arrest at the G1 phase [247,248,249].

CHOP, a downstream transcription factor of PERK, activates GADD34, which promotes the dephosphorylation of eIF2α and restores protein translation, creating a feedback loop that facilitates cell survival during acute ER stress. However, if the stress is chronic or excessively severe, the restored protein synthesis can be a double-edged sword, causing damage and ultimately triggering cell death [249,250,251].

Different pharmacological agents that can modulate PERK activity have been studied for their potential role in influencing tumor progression and response to treatment, including the following:

- GSK2606414 and GSK2656157: selective PERK inhibitors that suppress tumor growth in pancreatic, glioblastoma, and lung cancer models. However, systemic toxicity has limited their clinical application [252].
- ISRIB (integrated stress response inhibitor): a small molecule that restores protein synthesis by blocking eIF2α phosphorylation, reducing stress adaptation in colorectal and prostate cancer cells [253].
- Salubrinal: Initially developed as an eIF2α phosphatase inhibitor, salubrinal enhances ER stress and sensitizes tumor cells to chemotherapy in breast and liver cancer models [254].
- Metformin: Known for its metabolic effects, metformin inhibits PERK-Nrf2 signaling, reducing oxidative stress tolerance in hepatocellular cancer [255].
- Bortezomib: a proteasome inhibitor that induces excessive ER stress, leading to PERK-mediated apoptosis in multiple myeloma and leukemia [256].

## 4. The Interplay Between ER Stress and Cancer Metabolism

Cancer cells are characterized by dysregulated metabolism, which supports their rapid growth and survival in the challenging tumor microenvironment [219]. A critical aspect of this altered metabolism is the occurrence of ER stress, which arises when the ER protein folding capacity is overwhelmed, leading to the accumulation of misfolded proteins and the UPR. However, this latter is not only a response to protein misfolding but is also intricately linked to various metabolic pathways. ER stress influences the synthesis and utilization of key metabolites, including glucose, glutamine, amino acids, lipids, and nucleic acids. For instance, glucose dysregulation in the tumor microenvironment can exacerbate ER stress, as fluctuating glucose levels impair the synthesis of glycoproteins and disrupt redox balance within the ER lumen [17,18]. Glucose and glutamine are critical for cellular energy production, but under ER stress, their metabolism can be rerouted to support redox balance and biosynthetic processes, including protein and nucleic acid synthesis. Dysregulated glutamine metabolism can exacerbate ER stress, contributing to cell dysfunction and promoting disease progression [19]. For instance, increased glutamine flux can enhance the activity of the enzyme glutaminase, which produces glutamate, further driving the ER stress response [20]. Lipids, essential for membrane biosynthesis and signaling, are likewise impacted, as the UPR coordinates lipid metabolism to ensure cell membrane integrity and vesicular trafficking, which are critical for cell survival under stress. An excess of free fatty acids can contribute to the activation of the UPR pathway, specifically through the IRE1 and PERK sensors, which further propagate stress responses and impact cellular integrity [21]. Furthermore, the increased demand for nucleic acids during cell division and repair processes is regulated by UPR to support tumor cell proliferation. Recent studies have demonstrated a bidirectional relationship between nucleic acid damage and ER stress, suggesting that DNA damage can exacerbate UPR activation, while ER stress can further promote genomic instability [22].

In this context, metabolomics represents a strategic tool to provide a comprehensive view of metabolic alterations induced by endoplasmic reticulum stress, enabling the identification of diagnostic biomarkers and therapeutic targets. Integrated analysis of metabolic profiles allows for delineating specific metabolic signatures associated with endoplasmic reticulum stress in cancer cells, providing valuable insights into the functional status of cells and their adaptive strategies. This approach, combined with the study of UPR signaling pathways, provides a better understanding of the global impact of endoplasmic reticulum stress in tumor progression and response to treatments.

The dynamic interplay between ER stress and cancer metabolism modulates these networks, creating a favorable environment for tumor growth and survival. Below, a more detailed explanation is provided of the relationship between ER stress and the main biomolecules involved in cancer cell metabolism.

### 4.1. Glucose

The availability of glucose is closely linked to ER stress through multiple mechanisms [257].

A deficiency in glucose disrupts the hexosamine biosynthetic pathway (HBP), which relies on these nutrients to produce uridine diphosphate-N-acetylglucosamine (UDP-GlcNAc). This molecule is essential for N-linked glycosylation and proper protein folding within the ER [258,259].

Additionally, glucose scarcity impairs ATP production, which serves both as an energy source and a phosphate donor necessary for ER protein folding [260].

Reduced glucose availability also affects calcium homeostasis in the ER, largely through the reduced activity of the sarcoplasmic/endoplasmic reticulum calcium ATPase (SERCA) [261].

Under conditions of severe or prolonged ER stress, many signaling pathways can be activated, such as PERK-eIF2α, IRE1-XBP1, CHOP, mTOR, and AMPK, affecting cellular metabolism and leading to the upregulation of aerobic glycolysis [262].

Endoplasmic reticulum oxidoreductase 1 alpha (ERO1) is a flavin adenine nucleotide (FAD)-dependent luminal glycoprotein localized in the ER that promotes the formation of disulfide bonds in secretory and cell surface proteins [263,264]. Recent studies have shown that ERO1 is overexpressed in various human cancers, contributing significantly to several malignancy-associated traits of tumors, including enhanced growth, metastasis, angiogenesis, and immune evasion [265,266]. In this contest, sustained ER stress due to ERO1 overactivation results in chronic UPR activation, which enhances cell survival, proliferation, and metabolic reprogramming, particularly through the promotion of aerobic glycolysis [267]. Furthermore, ROS produced by ERO1 can activate HIF1α, which further promotes glycolytic metabolism, providing the tumor with the energy needed for rapid growth [268].

The increased ROS levels generated by ERO1 must be neutralized by reduced GSH to prevent cell death. Cancer cells typically maintain high GSH levels, utilizing NADPH indirectly generated by aerobic glycolysis, which feeds into the PPP pathway, to fuel this process [269]. In this context, augmented aerobic glycolysis may represent a cellular adaptation to UPR activation, enhancing cell survival and fitness.

Moreover, ERO1 has been implicated in the activation of various signaling pathways, including PI3K-Akt/mTOR, S1PR1/STAT3, and Wnt/β-catenin [270,271], which are known to drive rapid cell proliferation and contribute to the Warburg effect in cancer [272].

The role of glucose in the tumor microenvironment has been highlighted through the administration of many drugs such as versipelostatin (VST) to human HT-29 colon cancer, HT1080 fibrosarcoma, and MKN74 stomach cancer cells. VST exhibits a highly selective cytotoxic effect on tumor cells deprived of glucose, which is linked to the suppression of the UPR [273]. Specifically, the natural polyketide VST downregulates the expression of key UPR target genes, GRP78 and GRP94, inhibiting the production of UPR transcription factors XBP1 and ATF4, under glucose deprivation conditions. Furthermore, VST demonstrated in vivo antitumor activity at doses that were well tolerated in an MKN74 xenograft model, cultured in the absence of glucose [273].

Inhibition of glucose-regulated genes and their related pathways by versipelostatin disrupts key metabolic processes and enhances the vulnerability of cancer cells to stress and apoptosis. The PI3K/Akt/mTOR pathway plays a central role in regulating both metabolic processes and the activation of the UPR. This pathway is involved in the activation of HIF1α, inducing the expression of critical genes involved in glycolysis, including hexokinase 2 (HK2), phosphofructokinase 1 (PFK1), and LDHA, as well as glucose transporters such as GLUT1, facilitating enhanced glucose uptake and metabolism [274]. In addition to its indirect effects on glycolysis, versipelostatin also interferes with UPR, reducing the activation of UPR sensors, thereby compromising the cell’s ability to respond effectively to ER stress. In this way, the drug not only disrupts metabolic pathways but also impairs the cellular machinery that protects tumor cells from accumulating damage due to protein misfolding, increasing their susceptibility to apoptosis [275]. The combined inhibition of both glucose metabolism and UPR activation by versipelostatin forces tumor cells into a state of metabolic and proteotoxic stress. This significantly reduces their capacity to survive and proliferate, especially in environments where nutrient and oxygen availability is limited.

In addition, the disruption of these key processes can facilitate ROS accumulation, which further exacerbates cellular damage and contributes to the initiation of cell death pathways [276]. The ability of versipelostatin to amplify ROS levels and induce cell death is a potential therapeutic advantage, particularly when used in combination with other agents targeting specific vulnerabilities in cancer cells [277].

### 4.2. Fatty Acids

It is well established that lipid metabolism in tumor cells is closely associated with the IRE1 signaling pathway. For instance, in tumors driven by c-Myc, the IRE1α/XBP1s axis promotes tumor growth by enhancing the expression of stearoyl-CoA desaturase 1 (SCD1), a critical enzyme involved in lipid metabolism and tumor invasion within the ER [278,279]. Furthermore, studies have shown that IRE1α, through its role in RIDD, functions as a transcriptional repressor of the enzyme diacylglycerol O-acyltransferase 2 (DGAT2) in cells such as MDA-MB-231, HCC1806, and BT-549. This repression leads to a decrease in DGAT2 mRNA levels, subsequently lowering triacylglycerol accumulation in these cells [280]. In addition to the IRE1 pathway, lipid metabolism in tumors is also closely intertwined with the PERK pathway. Previous research has indicated that disruption of the PERK pathway results in reduced expression of key fatty acid synthesis enzymes, including fatty acid synthase (FASN), ACLY, and SCD1, thus linking PERK signaling with lipid metabolic regulation. Studies conducted using the HepG2 liver cancer cell model have demonstrated that ER stress induced by apoptin triggers alterations in lipid metabolism [281]. Specifically, this stress response leads to the upregulation of enzymes associated with lipid synthesis, such as FASN, ACC, phospholipase D1 (PLD1), and SCD1, within the first 24 h, followed by a subsequent decline in their expression [177,282]. Apoptin is particularly known for its ability to selectively induce apoptosis in tumor cells while leaving normal cells largely unaffected, making apoptin an attractive candidate for potential cancer therapies. Studies on HepG2 liver cancer cells have demonstrated that apoptin can cause an accumulation of misfolded proteins within the ER, activating UPR. Apoptin effectively induces ER stress in tumor cells, exploiting their disrupted protein homeostasis caused by mutations in regulatory pathways like p53. It selectively accumulates in the nucleus of cancer cells with mutated p53, causing nuclear damage and triggering apoptosis. In addition to inducing ER stress, the action of apoptin in cancer cells renders tumor cells more susceptible to death by interfering with their ability to maintain proper lipid metabolism [283]. A phenomenon frequently observed in various cancer cell types such as LNCaP androgen-dependent prostate cancer cells is the saturation of membrane phospholipids which disrupts the structural integrity of the ER [265] compromising its homeostasis [284,285]. Ariyama et al. showed that the knockdown of SCD1 increased the amount of saturated fatty acids and reduced that of monounsaturated fatty acids in phospholipids, inducing severe UPR activation, evidenced by increased expression of CHOP, GRP78, and XBP1 [286].

A similar fate in cancer cells is observed when the sterol regulatory element-binding protein (SREBP), a key transcriptional regulator of lipogenic genes, is inactivated in lipid-deprived conditions. This lipotoxic effect can be mitigated by the supplementation of exogenous unsaturated lipids [287] or by reintroducing SCD1 expression. Recent studies have highlighted that an imbalance in cholesterol homeostasis, specifically the accumulation of free cholesterol (FC), leads to ER stress in cancer cells. For instance, FC overload in HepG2 cells, induced by antitumor alkylphospholipids [288], such as perifosine, miltefosine, and edelfosine, was shown to trigger an upregulation of CHOP [289]. Similarly, inhibition of cholesterol esterification via targeting the enzyme sterol-O-acyltransferase 1 (SOAT1) was found to activate ER stress markers in adrenocortical adenocarcinoma cells [135,290]. Recent studies have demonstrated that lipid accumulation directly influences ER stress in cancer cells. In one experiment, HeLa cells were cultured under conditions that promoted lipid accumulation, such as high glucose and high serum. The results showed that these cells exhibited significantly increased lipid droplet formation and a concurrent rise in markers of ER stress, including CHOP and GRP78 [291]. Another experimental study using MCF-7 breast cancer cells showed that SREBP1 activation led to a significant increase in fatty acid synthesis, and this was associated with upregulated ER stress markers. Cells with enhanced fatty acid synthesis exhibited higher levels of ERAD machinery activity, indicating that the accumulation of misfolded proteins in the ER was a consequence of lipid overload [292]. Lipid metabolic reprogramming has also been shown to interact with other signaling pathways, such as PI3K/Akt and mTOR, which are frequently hyperactivated in cancer cells. These pathways modulate lipid biosynthesis and, when dysregulated, further exacerbate ER stress by increasing lipid synthesis beyond the ER’s capacity to process and export newly synthesized lipids [293].

A crucial aspect of cancer metabolism is lipotoxicity, a condition wherein excess fatty acids, particularly saturated ones, accumulate and damage cellular structures. This phenomenon is strongly associated with ER stress, as the excess lipids disrupt normal ER function and activate stress responses. Palmitic acid, a common saturated fatty acid, has been widely studied for its role in inducing lipotoxicity and ER stress in cancer cells [294]. Elevated levels of palmitate, stimulated by the SREBP1 pathway and alterations in the PI3K/Akt/mTOR signaling pathway, can promote the accumulation of misfolded proteins in the ER, triggering UPR activation, which may lead to either cell survival mechanisms or cell death. In addition to saturated fatty acids, ceramides have been shown to play a critical role in ER stress. Ceramides are sphingolipid metabolites that accumulate in response to lipotoxicity and act as second messengers in the signaling pathways leading to cell death. The accumulation of ceramides has been linked to ER stress and has been proposed as a diagnostic marker for various types of cancers, where they reflect the degree of metabolic stress and the lipotoxic environment within the TME. Another area of interest is the β-oxidation of fatty acids, a key process through which cells break down fatty acids to produce energy. In many cancers, β-oxidation is altered, either by upregulation to meet energy demands or by dysregulation that results in the accumulation of intermediate metabolites, such as acetyl-CoA and acyl-CoA. These intermediates, which are involved in energy production and lipid biosynthesis, can affect lipid homeostasis and contribute to ER stress. For instance, acetyl-CoA plays a central role in lipid biosynthesis and can influence the lipogenic pathways that are often upregulated in cancer cells [295]. Alterations in these pathways may not only support the tumor’s metabolic reprogramming but also exacerbate ER dysfunction and stress responses.

### 4.3. Amino Acids

Beyond glucose, limited amino acid availability represents another significant source of stress within the TME. Amino acid deprivation triggers the activation of the kinase GCN2, which phosphorylates eukaryotic translation initiation factor 2α. This modification initiates the integrated stress response (ISR), a critical adaptive mechanism that allows cancer cells to survive under nutrient-deprived conditions [296].

For instance, arginine availability is often limited due to metabolic alterations in the TME. Tumor cells frequently upregulate enzymes like arginase I, which catabolize arginine into ornithine and urea. This depletion of extracellular arginine can exacerbate ER stress in tumor cells [297]. The reduced availability of arginine impairs NO and polyamine synthesis, potentially hindering the cell’s ability to cope with the accumulation of misfolded proteins in the ER. As a result, arginine-deprived tumor cells may be more susceptible to apoptosis and may exhibit increased sensitivity to chemotherapeutic agents that further induce ER stress. Moreover, reduced arginine levels can affect immune cells within the TME. Arginine depletion has been shown to impair T-cell function and promote immune evasion in tumors, potentially contributing to an environment that favors tumor survival. Given the central role of arginine in maintaining cellular stress responses, supplementation with this amino acid has been proposed as a potential strategy to mitigate ER stress in tumors. Arginine supplementation may improve the folding capacity of the ER and enhance the ability of tumor cells to manage stress, thereby improving cell survival under challenging conditions [298]. However, the use of arginine supplementation in cancer therapy is not without challenges. While it may help alleviate ER stress and promote tumor cell survival, excessive arginine could also provide metabolic support that accelerates tumor growth. As such, the therapeutic use of arginine must be carefully managed to balance the benefits of stress alleviation with the risk of promoting tumor progression. In head and neck squamous cell carcinoma (HNSCC) cells, arginine depletion activates the UPR, a key pathway involved in mitigating ER stress. The depletion of arginine induced apoptosis through the activation of the eIF2α-ATF4(GADD34)-CHOP pathway, a hallmark of ER stress-induced cell death. Additionally, combining arginine deprivation therapy with canavanine, an arginine analog, resulted in enhanced radiosensitivity, suggesting that the metabolic manipulation of arginine levels can influence cancer treatment outcomes [299]. According to Miraki-Moud et al., arginine depletion using a pegylated form of arginine deiminase (ADI-PEG 20) induces antitumor activity on acute myeloid leukemia cells in a xenograft model and in vitro. The results showed increased expression of annexin-V and caspase 3/7 activity, supporting the idea that arginine depletion promotes cell death [300]. These findings are particularly relevant in preclinical cancer therapies targeting metabolic dependencies. The induction of apoptosis following arginine deprivation has been observed in breast cancer and lymphoma cell lines. In particular, in the MDA-MB-231 cell line, ADI-PEG 20 led to an increase in intracellular ROS, compromising mitochondrial integrity. This suggests that arginine deprivation may cause ER stress that contributes to cell death [301].

Alanine can be synthesized from pyruvate and is involved in various cellular functions, including protein synthesis, gluconeogenesis, and the maintenance of redox balance through its conversion to pyruvate. The alanine cycle, also known as the glucose–alanine cycle, facilitates the transfer of nitrogen between tissues and supports metabolic homeostasis, especially during conditions of high metabolic activity, such as those present in cancer [302]. The ability of alanine to modulate cellular metabolism and the redox state has critical implications for stress responses. Tumor cells often exhibit altered metabolic pathways to support their rapid proliferation, and the accumulation of metabolic byproducts can lead to ER stress. Alanine, as a key metabolic intermediate, may play a role in buffering this stress. Alanine metabolism can help sustain cellular energy levels and maintain a balance between oxidative and reductive reactions, which is essential for proper protein folding in the ER [303]. In tumor cells, the upregulation of alanine transferase, responsible for the conversion of pyruvate to alanine, is frequently observed and may contribute to enhanced alanine availability, allowing cells to better manage stress. Elevated levels of alanine can support energy metabolism through the alanine–glucose cycle, which can help alleviate oxidative stress and mitigate ER stress. Additionally, alanine’s role in the maintenance of mitochondrial function and ROS homeostasis further emphasizes its importance in regulating the cellular stress response [304]. In a study by Parker et al., the role of alanine transporters in PDAC cells was investigated, where they utilized a cDNA withdrawal approach to mimic the inhibition of the alanine transporter SLC38A2. The results demonstrated that the loss of alanine transport led to significant and durable tumor regression in fully formed tumors. This finding suggests that targeting alanine transport mechanisms could be an effective therapeutic strategy for pancreatic cancer [84,305]. Given the role of alanine in cellular stress responses, modulating its levels in the TME could represent a therapeutic strategy for managing ER stress in cancer cells. Enhancing alanine availability through supplementation may help tumors cope with metabolic challenges and reduce the accumulation of misfolded proteins in the ER, thereby alleviating ER stress. However, as with other amino acids, altering alanine metabolism in tumors could have complex effects. While alanine supplementation may enhance cell survival and support growth, it could also contribute to tumor progression by promoting metabolic flexibility, which could allow cancer cells to better adapt to hostile TME conditions [303]. On the other hand, inhibiting alanine synthesis or its metabolic pathway may impair tumor cell survival by exacerbating ER stress and compromising their ability to manage oxidative damage.

### 4.4. Nucleic Acids

In cancer cells, DNA damage is frequent due to the rapid proliferation, genomic instability, and exposure to genotoxic agents like chemotherapy and radiation. This damage can overwhelm the cell’s DNA repair mechanisms, leading to the activation of the DNA damage response (DDR). The DDR and UPR are not isolated pathways; they are interconnected and share several signaling molecules, particularly the kinase proteins that mediate cellular stress responses. One key example is PERK, which, upon activation by DNA damage, can also trigger UPR signaling by phosphorylating the eIF2α, which inhibits global protein translation and promotes the synthesis of stress-related proteins [306]. The connection between DNA damage and UPR activation in tumor cells is important because it highlights a feedback loop. While the UPR helps mitigate stress by improving protein folding and reducing the load on the ER, persistent or unresolved DNA damage can exacerbate ER stress, triggering an amplified UPR. In cancer, this can contribute to the acquisition of oncogenic traits, including resistance to apoptosis and enhanced survival under conditions that would normally induce cell death. Tumor cells often exploit this pathway to adapt to DNA damage, promoting tumorigenesis and therapy resistance [307].

In addition to DNA, RNA dysregulation in tumor cells also plays a major role in ER stress and UPR. Cancer cells often experience altered RNA metabolism as part of their metabolic reprogramming. The rapid proliferation and high demands for protein synthesis lead to a high burden on the ER, particularly in translating mRNAs into proteins. Abnormal or defective RNA transcripts, as well as increased transcriptional activity, can place additional stress on the ER’s ability to properly fold proteins, further triggering the UPR [308]. Furthermore, non-coding RNAs, including lncRNAs and miRNAs, have been shown to modulate the UPR and influence tumor cell survival. LncRNAs can interact with key components of the UPR machinery, regulating the activation of stress response proteins such as XBP1, which plays a crucial role in the UPR’s ability to mitigate cellular stress. These interactions may determine whether cells survive or undergo apoptosis in response to stress [309]. In a study with human lung cancer cells (A549), researchers induced DNA damage using gamma radiation and assessed the subsequent ER stress response. The results showed that DNA damage triggered the activation of the ER stress markers, such as CHOP, GRP78, and ATF4, indicating a shift in cellular homeostasis toward an ER stress response. Additionally, the expression of genes involved in the DNA damage response (like p53 and ATM) was upregulated, revealing a complex crosstalk between DNA damage and ER stress in the TME [310]. It was studied how RNA transcription is affected by ER stress. In HeLa cells, treated with tunicamycin, a known inducer of ER stress, the expression of XBP1 splicing variants was analyzed. The results showed that XBP1 splicing was significantly increased upon treatment. These findings suggest that nucleic acid splicing and transcription are directly impacted by ER stress and that XBP1 splicing could serve as a critical biomarker for ER stress in cancer cells [311]. MiRNAs, small non-coding RNAs that regulate gene expression post-transcriptionally, also play a critical role in UPR modulation in cancer. For instance, miR-146a is known to target components of the UPR signaling pathway and has been implicated in the regulation of cancer cell survival under stress conditions. Through the modulation of UPR-related genes, miRNAs can influence the balance between cell survival and cell death, providing cancer cells with an adaptive advantage [312]. The role of miRNAs in regulating ER stress was studied in an in vitro model of colorectal cancer (HCT116 cells). It was investigated how certain miRNAs, including miR-34a, modulate the ER stress response. miR-34a was found to inhibit the expression of ATF4, thereby reducing the extent of ER stress [313]. The accumulation of nucleic acids in the tumor microenvironment can exacerbate ER stress and UPR activation. In the context of tumor cell death, such as necrosis or apoptosis, nucleic acids are often released into the extracellular space. These extracellular DNA and RNA molecules can be recognized by pattern recognition receptors like Toll-like receptors (TLRs) on immune cells, further activating inflammatory pathways that can enhance ER stress within both tumor and immune cells [314]. This inflammatory response can contribute to a pro-tumorigenic environment, enhancing tumor growth and metastasis. Moreover, nucleic acids contribute to the complexity of the UPR through their interaction with the TME. Tumors are often characterized by a hypoxic microenvironment, which can lead to the accumulation of damaged RNA and altered splicing, further stressing the ER and leading to a heightened UPR response. This constant fluctuation between cellular stress and adaptive responses allows tumors to persist and evolve, even under unfavorable conditions (Table 2).

In Table 2, the main metabolic pathways involved in cancer, just described, are reported and are closely associated with ER stress.

## 5. ER Stress-Induced Metabolic Reprogramming and Its Impact on the Tumor Microenvironment and Cancer Resistance

Emerging evidence highlights that ER stress-induced metabolic reprogramming not only supports tumor growth and survival but also profoundly influences the composition and function of immune cells within the tumor microenvironment [315].

Macrophages play a key role in regulating innate immunity and activating T cells. Pro-inflammatory M1 macrophages enhance immune cell recruitment, while immunosuppressive M2 macrophages promote tissue repair. A dynamic balance between these two populations is essential for maintaining immune homeostasis, whereas its disruption is closely linked to tumor progression [316,317].

In particular, UPR signaling also influences macrophage recruitment and polarization, promoting the accumulation of immunosuppressive M2 macrophages. Preclinical studies have demonstrated that reducing GRP78 expression or inhibiting IRE1 in tumor cells enhances macrophage-mediated tumor cell clearance, while PERK inhibition promotes the expansion of M1 macrophages and strengthens the antitumor immune response. Overall, this evidence supports a dual mechanism in which tumor UPR promotes tumor survival while simultaneously suppressing macrophage-mediated immune responses [318].

For example, ER+ (estrogen receptor) breast cancer is the most common subtype of breast carcinoma, often treated with endocrine therapies such as tamoxifen. However, many patients develop resistance to these treatments, a phenomenon that has been associated with the overexpression of GRP78. Beyond its well-established role in regulating the ER stress response, GRP78 also plays a crucial role in controlling lipid metabolism in tumor cells. Its inhibition leads to the accumulation of essential fatty acids, indicating that GRP78 controls both lipid uptake and catabolism. Moreover, GRP78 depletion reduces the expression of SREBP-1, a key transcription factor involved in lipid biosynthesis.

At the same time, GRP78 inhibition reduces fatty acid β-oxidation by downregulating CPT1A, the key enzyme responsible for transporting fatty acids into mitochondria. The resulting lipid accumulation promotes lipid peroxidation and ROS production, contributing to lipotoxicity and sensitizing tumor cells to endocrine therapy. Additionally, the increased ROS levels and lipid stress caused by GRP78 silencing enhance the production of the chemokine MCP-1, which promotes macrophage recruitment within the tumor microenvironment.

Macrophage recruitment further enhances antitumor immune surveillance, facilitating the clearance of malignant cells. Consequently, combined treatment with tamoxifen and GRP78 inhibition in in vivo models of ER+ breast cancer not only restored tamoxifen sensitivity but also increased macrophage infiltration and lipid peroxidation, demonstrating the therapeutic potential of targeting GRP78 in endocrine-resistant breast tumors [319,320].

In a study by Soto-Pantoja et al. [321], specific RNAi were used to individually silence the distinct branches of the UPR (IRE1, PERK, and GRP78) to determine the effect of UPR inhibition on the plasticity of RAW 264.7 macrophages. The results showed that PERK inhibition increases macrophage proliferation, M1 polarization, and the ability to kill tumor cells. PERK normally limits the translation of pro-inflammatory cytokines, and its hyperactivation can induce macrophage apoptosis.

In contrast, IRE1 inhibition had no significant direct effect on macrophage polarization or cytotoxic activity. Finally, GRP78 inhibition promotes M2 polarization, reduces macrophage proliferation, and impairs their ability to eliminate tumor cells.

Inhibition of GRP78 and IRE1 in macrophages increases cellular lipid content, while PERK suppression favors glycolysis, ultimately promoting a pro-inflammatory M1 polarization. Macrophage polarization is tightly linked to metabolism: M1 macrophages primarily rely on glycolysis, whereas M2 macrophages depend on lipid β-oxidation [321].

Therefore, while GRP78 inhibition in macrophages shifts them toward an M2-like phenotype, this is driven by a compensatory increase in PERK and IRE1 signaling, which supports an M2-like profile. Notably, when GRP78 is inhibited, macrophages become less efficient at managing β-oxidation, but the lipid accumulation and residual metabolism still push them toward a metabolically and functionally M2-like state.

At the same time, GRP78 silencing in tumor cells, particularly in the murine breast cancer cell line 4T1B, leads to increased secretion of MCP-1, which recruits macrophages into the tumor microenvironment, and a reduction in CD47, a “self” signal that makes the tumor more vulnerable to macrophage-mediated phagocytosis [321,322].

A crucial and unexpected role of XBP1 has recently been uncovered in driving the dysfunction of tumor-associated dendritic cells (tDCs) within the tumor microenvironment. XBP1 is persistently activated in tDCs, disrupting their homeostasis and impairing their ability to present antigens, thereby fostering local immunosuppression and contributing to immune evasion by tumor cells.

tDCs accumulate abnormal levels of oxidized lipids, a process directly promoted by XBP1 and byproducts of lipid peroxidation, such as the reactive aldehyde 4-hydroxy-trans-2-nonenal (4-HNE). These reactive metabolites not only perpetuate ER stress but also sustain chronic activation of the IRE1α/XBP1 pathway in tDCs, blocking their immunostimulatory function and contributing to an immunosuppressive state that impairs antitumor T-cell activation [323,324,325].

This pathological cascade is particularly relevant in patients with ovarian cancer, where persistent oxidative stress and 4-HNE generation, further amplified by chemotherapy, promote chronic ER stress in infiltrating immune cells, ultimately contributing to disease recurrence.

In a study by Cubillos-Ruiz et al., selective elimination of XBP1 in tDCs, using siRNA-loaded nanocarriers, reversed the immunosuppressive phenotype of tDCs, converting them into cells capable of mounting a strong antitumor immune response and significantly improving survival in preclinical models. This strategy paves the way for new immunotherapies targeting XBP1, with the potential to improve prognosis in ovarian cancer and other aggressive tumors, by simultaneously reducing tumor cell survival and restoring an effective antitumor immune microenvironment [326].

Moreover, the UPR activates the secretion of pro-inflammatory cytokines and chemokines, such as IL-6 and IL-8, which promote local chronic inflammation and promote the recruitment of immune cells with pro-tumor phenotypes, such as tumor-associated macrophages with immunosuppressive characteristics [327]. Notably, the IRE1α-XBP1 signaling pathway has also been linked to tumor progression and therapy resistance, in part through the positive regulation of IL-6 in several types of cancer, including hepatocellular carcinoma and melanoma. Elevated IL-6 expression driven by XBP1s promotes tumor cell proliferation through activation of the STAT3 signaling pathway [328,329].

Similar to macrophages, T cell metabolism is tightly regulated by nutrient availability and stress signals within the tumor microenvironment. ER stress can impair T cell effector function by inducing a metabolic shift from glycolysis to fatty acid oxidation, which is associated with exhaustion-like phenotypes in cytotoxic T lymphocytes (CTLs) and reduced antitumor immunity [330].

The IRE1α-XBP1s signaling within the UPR negatively impacts intratumoral CD8+ T cells, limiting their antitumor function through several metabolic mechanisms. In the tumor microenvironment, CD8+ T cells display reduced expression of the glucose transporter GLUT1, which hampers glucose uptake and compromises glycolysis, a critical process for their activation and effector function. This reduction in glucose uptake disrupts T cell mitochondrial metabolism, making them less efficient at producing energy and limiting their ability to mount effective antitumor responses.

Furthermore, XBP1s activation reduces the expression of glutamine transporters, preventing T cells from utilizing glutamine as an alternative energy source. This results in mitochondrial dysfunction, further impairing CD8+ T cell efficacy against tumors.

Another detrimental effect of XBP1s hyperactivation in CD8+ T cells is the upregulation of inhibitory immune checkpoints such as programmed cell death protein 1 (PD-1) and signaling lymphocytic activation molecule family member 4 (2B4), further contributing to the functional exhaustion (“T cell exhaustion”) typical of T cells in tumors.

In addition, the accumulation of cholesterol within the TME exacerbates this dysfunction, fostering an immunosuppressive environment. Consequently, targeted inhibition of the IRE1α-XBP1s pathway in T cells has been shown to enhance their antitumor activity and prolong survival in certain cancer models, including murine melanoma models. This highlights the potential of targeting this metabolic pathway as a promising strategy to enhance cancer immunotherapy [331,332].

### 5.1. ER Stress-Driven Metabolic Reprogramming Promotes Cancer Stemness and EMT

Cancer cells exhibit remarkable phenotypic plasticity, which contributes to intratumoral heterogeneity. This plasticity allows differentiated cancer cells to undergo retrodifferentiation, reverting to a more immature state and acquiring features typical of cancer stem cells (CSCs) [333]. Cancer stem cells are therefore a subpopulation of malignant cells with a high capacity for self-renewal and resistance to treatments. This transition is closely linked to metabolic reprogramming, and according to the “metabostemness” theory, metabolic reprogramming represents one of the earliest events in the acquisition of stem-like properties and the activation of epithelial-to-mesenchymal transition. CSCs frequently adopt a glycolytic metabolism, despite retaining functional mitochondria, thereby reducing the production of ROS and protecting themselves from oxidative damage. However, depending on the tissue and microenvironmental context, some CSCs prefer oxidative phosphorylation, while others adopt a hybrid metabolic profile [334,335].

EMT is a fundamental biological process through which epithelial cells lose their characteristic adhesion and polarity to acquire migratory and invasive properties. This process is orchestrated by EMT-specific transcription factors (EMT-TFs) [336,337], such as zinc finger E-box binding homeobox 1 (ZEB1), Snail family transcriptional repressor 1 (Snail) [338], and Snail family transcriptional repressor 2 (Slug) [339], and is associated with the acquisition of stemness markers such as CD44, CD133, and Nanog. During EMT, cancer cells acquire mesenchymal traits and enhanced survival capacity under microenvironmental stress conditions, making them more resistant to cell death and conventional therapies. Moreover, EMT is often incomplete in cancer cells, generating intermediate (quasi-mesenchymal) states characterized by high plasticity and the ability to colonize new metastatic sites.

Metabolic reprogramming is both a consequence and a driver of EMT and cancer stemness. EMT transcription factors such as Snail and ZEB1 directly regulate key glycolytic enzymes and suppress mitochondrial activity, favoring the metabolic shift toward aerobic glycolysis. At the same time, this metabolic alteration can induce phenotypic plasticity and promote EMT [340]. For example, increased glycolytic activity and lactate production can activate autocrine and paracrine signals that trigger EMT and enhance the acquisition of stem-like properties. Indeed, the upregulation of glycolytic enzymes such as hexokinase 2 and lactate dehydrogenase A, together with increased lactate production, is directly involved in EMT induction [341,342].

Alternative metabolic pathways, such as glutaminolysis, also play a key role in supporting CSC plasticity by providing metabolic intermediates for macromolecule synthesis and enhancing oxidative stress defense. In particular, glutaminase activity is frequently upregulated in EMT-driven tumors, supporting both energy production and redox homeostasis. Moreover, certain Krebs cycle intermediates, such as succinate and fumarate, act as true oncometabolites, epigenetically regulating EMT by inhibiting key demethylases [343,344].

Lipid metabolism is also closely linked to EMT. Cancer cells actively regulate fatty acid synthesis and cholesterol metabolism, thereby altering membrane fluidity and surface signaling that sustain invasion and metastasis. Cholesterol accumulation and lipid raft disruption have been directly linked to EMT, highlighting potential therapeutic targets such as FASN and cholesterol-lowering drugs. Lipid droplet accumulation and lipophagy activation further enhance CSC adaptation to adverse metabolic conditions [345,346].

Finally, the inflammatory tumor microenvironment and neuroendocrine signals, such as catecholamines, contribute to integrating metabolic reprogramming with phenotypic plasticity. Catecholamines (epinephrine and norepinephrine) promote EMT factor expression, glycolysis, and lactate synthesis through β2-adrenergic receptor activation, thus enhancing the acquisition of stem-like traits and therapy resistance [347]. This tight link between metabolism, EMT, and stemness makes ER stress-associated metabolic reprogramming a promising therapeutic target to counteract tumor progression and therapy resistance [348,349].

Pro-inflammatory cytokines such as TGF-β1, IL-6, and TNF-α promote both EMT and metabolic reprogramming, further contributing to CSC formation. At the molecular level, TGF-β1 activates the EMT transcription factors Snail, Slug, and ZEB1, which repress epithelial genes and activate mesenchymal genes, promoting phenotypic transition [350,351] IL-6 activates the JAK/STAT3 pathway, which not only regulates EMT factor transcription (including Snail and ZEB1) but is also essential for CSC survival and self-renewal [352,353]. TNF-α promotes EMT through NF-κB pathway activation, which in turn enhances Snail and Slug expression [354].

In particular, ZEB1 directly represses E-cadherin by binding to E-box elements in its promoter, causing loss of adhesion and acquisition of a mesenchymal phenotype. ZEB1 overexpression has been linked to tumor progression and metastasis in various cancers, including breast and colorectal cancer. Slug and Snail also play critical roles in repressing E-cadherin and promoting cell motility. Activation of these transcription factors also increases the expression of mesenchymal proteins such as vimentin and N-cadherin.

IL-6 and TNF-α enhance glucose uptake and aerobic glycolysis (Warburg effect) to support biosynthesis and ATP generation under stress conditions [355]. TGF-β1 promotes a hybrid metabolic profile in EMT-like cells, enhancing both glycolysis and lipid dependence [356,357]. Pro-inflammatory cytokines also regulate amino acid metabolism, increasing dependence on glutaminolysis, which is essential for nucleotide synthesis and redox balance [358].

Cancer stem cells are strongly influenced by the activation of the UPR. In solid tumors, PERK and IRE1 signaling pathways have been shown to play a crucial role in maintaining stemness and regulating cell fate. In particular, the IRE1-XBP1 axis acts as a key regulatory mechanism driving EMT by promoting the expression of mesenchymal transcription factors, such as Slug and Snail [359]. Moreover, EMT increases the sensitivity of cells to ER stress, triggering the PERK-eIF2α branch of the UPR, thereby creating a feedback loop that further enhances the mesenchymal transition [360]. In addition, PERK promotes both migration and invasion of lung cancer cells in vitro and in vivo models [361]. In colorectal cancer cells, ER stress has been directly linked to the induction of ZEB1 (zinc finger E-box binding homeobox 1). In the absence of ZEB1, colorectal cancer cells are unable to initiate ER stress responses following stimuli from the tumor microenvironment [362].

The UPR influences various aspects of tumor progression, including EMT and CSC differentiation and quiescence [363,364]. For example, the activities of PERK and XBP1 regulate lipid and amino acid metabolism, providing essential substrates for CSC growth and survival.

Under stress conditions, particularly during hypoxia or nutrient deprivation, PERK activation promotes a metabolic shift toward aerobic glycolysis, even in the presence of oxygen. This metabolic adaptation is critical for CSCs, as glycolysis allows for the rapid production of energy, the supply of metabolic intermediates needed for the biosynthesis of nucleotides, amino acids, and lipids, and the reduction of reactive oxygen species levels, enhancing CSC survival in hostile environments [365].

The PERK-ATF4 axis not only regulates glycolysis but also plays a fundamental role in amino acid metabolism, particularly glutaminolysis. ATF4 controls the expression of key enzymes involved in glutamine metabolism, which provides precursors for glutathione synthesis, essential for redox balance maintenance and protecting CSCs from oxidative stress.

The IRE1α-XBP1 branch also regulates de novo lipogenesis and cell membrane remodeling, which are essential processes for CSCs during EMT, as they require constant lipid reprogramming to support their functional activities.

CSCs exposed to microenvironmental stress rely on the UPR to rapidly shift between glycolysis and oxidative phosphorylation, exploiting their metabolic plasticity to adapt to changing conditions. This adaptability is enabled by the dual function of the UPR, which can activate catabolic pathways to survive nutrient deprivation and anabolic pathways to sustain proliferation when favorable conditions return.

A study by Mao et al. [366] demonstrated that activation of the IRE1α-XBP1 pathway is directly involved in metabolic reprogramming and in the induction of EMT in NSCLC cells. Using thapsigargin to induce acute ER stress, the study observed a significant upregulation of PDK1 (Pyruvate Dehydrogenase Kinase 1), a key metabolic regulator that shifts pyruvate metabolism away from oxidative phosphorylation toward aerobic glycolysis, thereby promoting lactate production. The accumulation of lactate in the tumor microenvironment not only facilitates cell migration and invasion but also directly induces Snail expression, mediated by the activation of the TGF-β pathway. The direct binding of XBP1 to the PDK1 promoter was confirmed, demonstrating that XBP1s acts as a transcriptional regulator of PDK1 in response to ER stress.

This strong dependence of CSCs on UPR-mediated metabolic control makes them particularly vulnerable. Selective inhibitors of PERK, IRE1α, or XBP1, when combined with metabolic-targeted therapies (such as glycolysis or glutaminase inhibitors), could selectively target CSCs, enhancing the effectiveness of standard therapies.

### 5.2. ER Stress-Associated Metabolic Reprogramming and Cancer Cell Resistance

Cancer resistance to chemotherapy and radiotherapy is closely linked to metabolic reprogramming, which enables tumor cells to adapt to stress, evade cell death, and sustain energy production. Altered metabolism supports drug resistance by enhancing antioxidant defenses, promoting DNA repair, and modulating the tumor microenvironment.

It has been established that, just as ER stress plays a crucial role in shaping cancer cell metabolism, this effect can also significantly contribute to therapy resistance.

For example, the PERK pathway represents a key element in the tolerance of therapy-resistant cells that emerge in response to transient changes in oxygen availability in solid tumors. Using isogenic models, it has been demonstrated that the eIF2α-dependent branch of the UPR promotes cysteine uptake and glutathione synthesis in hypoxic cells directly involved in tumor radioresistance, thereby strengthening antioxidant defenses and reducing susceptibility to oxidative damage induced by chemotherapy [367,368].

The PERK-Nrf2 signaling axis is closely linked to multidrug resistance in de-differentiated cancer cells, which are often associated with poor prognosis. This has been demonstrated using isogenic pairs of human breast epithelial cells (HMLE), which, unlike differentiated tumor cells, where Nrf2 activation is typically a response to oxidative stress via the PERK-mediated UPR pathway, exhibit constitutive PERK-Nrf2 signaling. This persistent activation drives the expression of antioxidant genes even before oxidative stress occurs, equipping the cells with intrinsic resistance to chemotherapy [369,370].

The IRE1-XBP1 signaling pathway, which promotes lipid remodeling and the synthesis of membrane components essential for ER expansion, supports both stress adaptation and cell survival under radiotherapy-induced damage [371]. Following acute ER stress experimentally induced by thapsigargin on two human lung adenocarcinoma cell lines (A549 and H1299), significant activation of IRE1α is observed, along with the consequent non-conventional splicing of XBP1. This transcription factor enhances the expression of SREBP1, a key regulator of lipid metabolism, which in this context takes on an additional role: SREBP1 directly binds to the promoter of MRP1, an efflux pump belonging to the ABC transporter family, increasing the transcription of ABCC1, the gene encoding MRP1. This protein actively exports numerous cytotoxic drugs from cancer cells, thereby reducing intracellular drug accumulation and limiting chemotherapy efficacy [372].

Moreover, the metabolic crosstalk between cancer cells and the tumor microenvironment, influenced by ER stress, promotes lactate secretion, immunosuppression, and resistance to immunotherapy [373]. These metabolic adaptations not only provide cancer cells with the flexibility to survive cytotoxic treatments but also promote pro-tumoral inflammation and extracellular matrix remodeling, further reducing the efficacy of both chemotherapy and radiotherapy. Targeting ER stress-associated metabolic reprogramming, either by inhibiting key branches of the UPR or exploiting specific metabolic vulnerabilities, is therefore emerging as a promising strategy to enhance the effectiveness of conventional anticancer therapies.

## 6. Conclusions

Endoplasmic reticulum stress occurs when the ER is unable to properly fold proteins, leading to the accumulation of misfolded proteins. In response, the cell activates the UPR, an adaptive mechanism aimed at restoring protein homeostasis. In cancer, UPR plays a paradoxical role, promoting tumor cell survival in stressful environments and tumor progression, while also potentially activating pro-death mechanisms, either dependent or independent of UPR. This complex cellular pathway, mainly regulated by sensors such as IRE1, PERK, and ATF6, performs distinct functions in modulating tumor survival and development, promoting or inhibiting tumor growth depending on the cancer type.

A crucial aspect of tumor cell survival is the production of sufficient metabolic energy for cellular repair and membrane potential maintenance. However, the metabolic changes induced by ER stress and their impact on cellular fate decisions remain largely unknown. This review discusses the correlations between UPR and metabolites involved in tumor onset and progression. Alterations in these metabolites reflect fundamental changes in cellular metabolism that support tumor growth and treatment resistance. Monitoring these metabolites could help identify high-risk patients, predict treatment outcomes, and optimize therapeutic strategies. Metabolomics thus represents a promising tool for supporting the discovery of new anti-cancer therapeutic strategies.

UPR is closely connected to various metabolic pathways, influencing the synthesis and use of key metabolites such as glucose, glutamine, lipids, and nucleic acids, which are essential for cell proliferation and DNA repair. Glucose availability is closely linked to ER stress, as its deficiency disrupts crucial metabolic pathways like the hexosamine pathway and ATP production, both essential for proper protein folding in the ER. Glucose deficiency activates pathways such as PERK and mTOR, promoting aerobic glycolysis. Drugs like versipelostatin inhibit the glucose stress response, compromising tumor cell survival.

Amino acid depletion, such as arginine, activates the integrated stress response, helping tumor cells survive under nutrient deprivation but may also worsen ER stress and increase sensitivity to chemotherapy. Alanine, by modulating the redox balance, supports the management of ER stress in tumor cells. However, altering arginine or alanine levels could have contrasting effects, enhancing cell survival but also favoring tumor progression. Additionally, fatty acid metabolites are crucial in connecting lipid metabolism to ER stress in tumor cells. Alterations in lipid composition, such as the accumulation of saturated fatty acids, disruption of fatty acid desaturation, and increased lipotoxicity, are associated with ER dysfunction and cancer progression. Other lipid metabolites, like ceramides, which accumulate in response to lipotoxicity, play a key role in activating ER stress.

The dynamic interactions between ER stress and tumor metabolism largely create an environment conducive to tumor growth, suggesting that monitoring metabolites associated with cellular metabolism and ER stress, such as lactate, pyruvate, glucose, and glutamine, could serve as predictive markers for diagnosis, prognosis, and treatment response in tumors, opening new possibilities for personalized oncology treatments.

## Figures and Tables

**Table 1:** Key metabolic changes and biomarkers.

Metabolic Change	Biomarkers	References
Increased glycolysis (Warburg effect)	Lactate, Pyruvate, GLUT1	[48,54,55]
Altered glutamine metabolism (glutaminolysis)	Glutamine, Glutamate, α-ketoglutarate	[61,62,63,64,65,66]
Lipid remodeling (phospholipid synthesis)	Phosphatidylcholine, Sphingomyelin	[125,126,127,128]
Increased oxidative stress response	Glutathione, NADPH	[57,66]
Altered amino acid metabolism	Alanine, Arginine, Carnitine	[80,81,82,83,84,89,90,91,92,93,94,95,96]
Increased nucleotide biosynthesis	Purines (IMP, AMP, GMP), Hypoxanthine	[155,156,157,158,159,160]
Increased ketone body metabolism	β-hydroxybutyrate, Acetoacetate	[145,146,147,148]
Altered folate metabolism	5-MTHF, Homocysteine	[162,163,164,165,166,167,168,169]
Cholesterol and sterol metabolism	Cholesterol, SREBP1, PCSK9	[135,136,137,138,139,140,141,142,143,144]

**Table 2:** Main metabolites involved in cancer, which play a role in ERS.

Metabolites	Pathway	Role in Cancer	Role in Ers	References
Glucose	Aerobic glycolysis	Warburg effect facilitates rapid tumor growth. ATP production.	Increased aerobic glycolysis in cancer cells leads to higher levels of ROS, promoting reticulum stress.	[260,262,268]
	Glucose deprivation	Tumor cells are resistant to glucose deprivation, maintaining survival through metabolic adaptations.	Glucose scarcity: Impairs ATP production, affecting protein folding and ER function. Impairs SERCA activity, disrupting calcium homeostasis and contributing to ER stress.	[261,273]
	Endoplasmic reticulum oxidoreductase 1 alpha (ERO1)	Overexpression of ERO1 enhances tumor growth, metastasis, and immune evasion.	ERO1 overactivation leads to chronic UPR activation, supporting cell survival and metabolic reprogramming.	[263,265,266]
	Hexosamine biosynthetic pathway (HBP)	UDP-GlcNAc is crucial for protein glycosylation and cell surface signaling.	Glucose deficiency disrupts the HBP, impairing N-linked glycosylation and protein folding in the ER.	[257,258,259]
**Fatty Acids**				
Phospholipids	Enhanced mitochondrial activity	Proliferation of tumor cells.	The accumulation of phospholipids in cell membranes can impair ER function by activating ER stress.	[129,283,284,291]
Acetyl-CoA	β-oxidation of fatty acids	Alterations in β-oxidation of fatty acids are implicated in supporting tumor growth.	Alteration in β-oxidation of fatty acids and accumulation of intermediate metabolites such as acetyl-CoA may contribute to ER stress.	[295]
Saturated fatty acids Cholesterol	SREBP1, PI3K/Akt, mTOR	Contribute to lipotoxicity in cancer cells by damaging cell structures.	Excess saturated fatty acids (such as palmitate) induce ER stress, accumulating misfolded proteins and activating the UPR.	[294,295]
SCD1 (stearoyl-CoA desaturase 1)	SREBP1, PI3K/Akt, mTOR	SCD1 promotes the synthesis of unsaturated fatty acids, increasing tumor proliferation and invasiveness.	SCD1 regulates lipid metabolism in the ER, contributing to lipid homeostasis and ER stress.	[278,279]
Free cholesterol (FC)	SREBP2, PI3K/Akt, mTOR	FC accumulation in tumor cells promotes invasion and metastasis.	FC overload causes ER stress, activating markers such as CHOP.	[288,289]
Phosphatidylserine (PS) Fosfatidilcolina (PC)	Sphingolipid metabolism, Choline metabolism, SREBP1	PS and PC contribute to membrane integrity and invasiveness of cancer cells.	Altered PS and PC in membranes can reduce ER stability, contributing to stress.	[283]
**Amino Acids**				
Alanine	TCA cycle	Enhance cancer cell growth because it acts as an anaplerotic substrate, replenishing the TCA cycle in cancer cells.	Helps to sustain cellular energy levels and maintains a balance between oxidative and reductive reactions essential for folding in the ERs.	[80,82,303,304]
	Nucleotide production	Enhance cancer cell growth.	Contributes to tumor progression by promoting metabolic flexibility.	[80,303,304,305]
	Glucose–alanine cycle	It serves as a nitrogen carrier and a gluconeogenic substrate, important in the metabolic reprogramming of cancer cells.	Elevated levels of alanine can support energy metabolism, which can help alleviate oxidative stress and mitigate ER stress.	[81,304]
Arginine	Polyamine synthesis	Rapid proliferation.	Arginine depletion can exacerbate ER stress in tumor cells.	[91,297]
	NO synthase	NO promotes cancer development, suppresses T-cell activation, and induces genomic instability interfering with the DNA repair mechanism.	Arginine-deprived tumor cells may be more susceptible to apoptosis and may exhibit increased sensitivity to chemotherapeutic agents that further induce ER stress.	[97,99,298]
Nucleic acids	ExDNA	Promote tumor cell adhesion, migration, and metastasis. exDNA induces pro-inflammatory cytokines and enhances tumor-promoting inflammation.	Persistent or unresolved DNA damage can exacerbate ER stress, triggering an amplified UPR.	[149,150,307]
	ExRNA	Regulate gene expression cells, influencing angiogenesis, immune evasion, and metastasis.	Abnormal or defective RNA transcripts can place additional stress on the ER’s ability to properly fold proteins, further triggering the UPR.	[151,308]

## Data Availability

No new data were created or analyzed in this study.
